# Downstream components of the calmodulin signaling pathway in the rice salt stress response revealed by transcriptome profiling and target identification

**DOI:** 10.1186/s12870-018-1538-4

**Published:** 2018-12-05

**Authors:** Worawat Yuenyong, Aumnart Chinpongpanich, Luca Comai, Supachitra Chadchawan, Teerapong Buaboocha

**Affiliations:** 1 Department of Biochemistry, Faculty of Science, Chulalongkorn University, Bangkok, Thailand; 2 Department of Plant Biology and Genome Center, University of California Davis, Davis, CA 795616 USA; 3 Center of Excellent in Environment and Plant Physiology, Department of Botany, Faculty of Science, Chulalongkorn University, Bangkok, Thailand; 4 Omics Sciences and Bioinformatics Center, Faculty of Science, Chulalongkorn University, Bangkok, Thailand

**Keywords:** Calmodulin, CaM, Rice, Salt stress, Transcriptome

## Abstract

**Background:**

Calmodulin (CaM) is an important calcium sensor protein that transduces Ca^2+^ signals in plant stress signaling pathways. A previous study has revealed that transgenic rice over-expressing the calmodulin gene *OsCam1–1* (LOC_Os03g20370) is more tolerant to salt stress than wild type. To elucidate the role of *OsCam1–1* in the salt stress response mechanism, downstream components of the *OsCam1–1*-mediated response were identified and investigated by transcriptome profiling and target identification.

**Results:**

Transcriptome profiling of transgenic ‘Khao Dawk Mali 105’ rice over-expressing *OsCam1–1* and wild type rice showed that overexpression of *OsCam1–1* widely affected the expression of genes involved in several cellular processes under salt stress, including signaling, hormone-mediated regulation, transcription, lipid metabolism, carbohydrate metabolism, secondary metabolism, photosynthesis, glycolysis, tricarboxylic acid (TCA) cycle and glyoxylate cycle. Under salt stress, the photosynthesis rate in the transgenic rice was slightly lower than in wild type, while sucrose and starch contents were higher, suggesting that energy and carbon metabolism were affected by *OsCam1–1* overexpression. Additionally, four known and six novel CaM-interacting proteins were identified by cDNA expression library screening with the recombinant OsCaM1. GO terms enriched in their associated proteins that matched those of the differentially expressed genes affected by *OsCam1–1* overexpression revealed various downstream cellular processes that could potentially be regulated by OsCaM1 through their actions.

**Conclusions:**

The diverse cellular processes affected by *OsCam1–1* overexpression and possessed by the identified CaM1-interacting proteins corroborate the notion that CaM signal transduction pathways compose a complex network of downstream components involved in several cellular processes. These findings suggest that under salt stress, CaM activity elevates metabolic enzymes involved in central energy pathways, which promote or at least maintain the production of energy under the limitation of photosynthesis.

**Electronic supplementary material:**

The online version of this article (10.1186/s12870-018-1538-4) contains supplementary material, which is available to authorized users.

## Background

Salinity stress is a major abiotic stress that affects plant growth, resulting in a loss of crop yield, especially rice, which is one of the most salt-sensitive plants in comparison to other cereals [1]. Salt stress affects plants via both osmotic and ionic effects. Osmotic effects result in a reduction of water absorption ability such that the effects are similar to drought stress. Ionic stress causes Na^+^ toxicity, which disrupts photosynthesis, protein synthesis, and enzyme activity [2, 3]. Numerous reports have shown negative effects of salt stress on rice growth and productivity based on the total chlorophyll content, protein concentration [4], photosynthetic CO_2_ fixation, stomatal conductance, transpiration [5], shoot dry weight, tiller number per plant, spikelets per panicle, and grain yield [6].

Ca^2+^ is a crucial second messenger consisting of a transient elevation of cytosolic [Ca^2+^]. The Ca^2+^ signals are transduced and decoded via Ca^2+^ binding protein, and then the information is relayed to downstream responses. The signals are mainly transduced through kinases mediating the phosphorylation cascade, resulting in downstream response regulation, including changes in gene expression through the regulation of transcription factors [7]. Calcium signaling is used to respond to environmental stimuli, as well as to coordinate growth and development in plants. In the plant calcium signal transduction process, calcium sensors, including calmodulin (CaM), calcineurin B-like (CBL) protein and Ca^2+^-dependent protein kinase (CPK), play important roles in the transduction of various stimuli [8, 9].

CaM is a protein that contains characteristic EF-hand motifs that bind Ca^2+^ ions with high affinity and specificity [10]. CaM binding to Ca^2+^ leads to the exposure of hydrophobic regions on the molecule surface and subsequent interactions with target proteins or nucleic acids [11]. Rice carries 5 CaM-encoding genes: *OsCam1–1*, *OsCam1–2*, *OsCam1–3*, *OsCam2* and *OsCam3* [12]. The expression of *OsCam1–1* increases to a great extent in response to NaCl, mannitol and wounding treatment [13]. Several lines of evidence have revealed that calcium sensors are involved with an enhanced abiotic tolerance capacity in plants [14]. Evidence has shown that the constitutive expression of bovine calmodulin in tobacco results in a shortened germination time of transgenic tobacco seeds under salt stress (120–160 mM NaCl) [15]. Arabidopsis overexpressing *GmCaM4* (*Glycine max* calmodulin) exhibit increased expression of AtMYB2-regulated genes, including proline-synthesizing enzymes, suggesting that this feature confers salt tolerance to the transgenic Arabidopsis by enabling the accumulation of proline [16]. Our previous report have shown that transgenic rice over-expressing *OsCam1–1* grow better under salt stress than wild type [17]. Wu H. and colleagues have found that the biphasic Ca^2+^ signal and enhancement of *OsCam1–1* expression in rice cause heat stress-mediated expression of downstream heat shock-related genes, and *OsCam1–1* overexpression Arabidopsis are more tolerant to heat stress than its wild type [18]. In another report, *AtCam3* knockout mutant Arabidopsis showed a clear reduction of thermotolerance after heat treatment at 45 °C, and when *AtCam3* was overexpressed in mutant and wild type Arabidopsis, the thermotolerant ability was rescued and increased, respectively. Moreover, co-expression of some heat shock protein genes with *AtCaM3* suggested that *AtCam3* plays a key role in the Ca^2+^-CaM heat shock transduction pathway [19]. The versatile functions of CaM are interesting, especially the role in the regulation of gene expression. CaM proteins directly modulate transcription factors (TFs), and some of these TFs have been verified to play roles in stress signaling pathways; however, the Ca^2+^ and Ca^2+^/CaM-regulating TF mechanisms remain incompletely understood and require further investigation [20, 21]. Transcriptomics analysis can lead to the discovery of genes or processes that respond to such factors. The aim of the present study was to investigate the downstream effects of *OsCam1–1* overexpression on gene expression regulation in rice under salt stress using a transcriptomic approach and to identify the interacting proteins to elucidate the role of *OsCam1–1* in the salt stress response mechanism.

## Results

### RNA-Seq of Rice overexpressing OsCam1–1 and differential gene expression analysis

CaM is a multifunctional protein that regulates the activities of numerous target proteins. Genome-wide analysis techniques such as transcriptome profiling are particularly suitable for identifying the downstream components that are potentially regulated by CaM. In our previous report [17], rice overexpressing *OsCam1–1* showed a significantly higher relative growth rate than wild type when grown under salt stress. Here, transcriptome profiling of the 3-week-old rice leaves of transgenic rice over-expressing *OsCam1–1* (L1) and its wild type (WT) under normal condition (NS) and salt stress (150 mM NaCl) conditions (S) for 4 h was conducted. More than 185 million reads from eight libraries from single-end RNA-Seq by Illumina Hi-Seq 2000 were obtained, with a total read of each library between 22 and 25 million reads. The reads were processed by POPE [22], which provided a total clean read per library of more than 99% of the total reads. At least 93% of the clean reads were mapped to the rice genome reference, Michigan State University rice annotation project’s MSU7 [23] and less than 11% of the clean reads were multiple alignment reads (Table 1).

**Table 1:** RNA-Seq read count information

Sample^a^	Raw Input Reads	Clean Reads	% Clean Reads	Mapped Reads	% Mapped Reads^b^	Multiple Alignment Reads	%Multiple Alignment Reads^b^
WTNS R1	24,019,397	23,961,896	99.76	22,973,989	95.88	1,952,214	8.15
WTNS R2	22,859,782	22,779,097	99.65	21,709,287	95.30	2,029,217	8.91
WTS R1	23,050,208	22,990,113	99.74	21,979,140	95.60	1,746,186	7.60
WTS R2	23,437,864	23,362,805	99.68	22,119,198	94.68	2,102,270	9.00
L1NS R1	23,259,980	23,234,303	99.89	22,207,203	95.58	2,048,681	8.82
L1NS R2	23,944,162	23,859,454	99.65	22,673,161	95.03	2,110,311	8.84
L1S R1	22,358,313	22,282,400	99.66	21,248,337	95.36	2,332,891	10.47
L1S R2	22,980,550	22,928,854	99.78	21,527,676	93.89	1,739,326	7.59

^a^ R1 and R2 indicate biological replicates

^b^The % mapped reads and % multiple alignment reads were calculated using the clean reads as a denominator

**Fig. 1:**
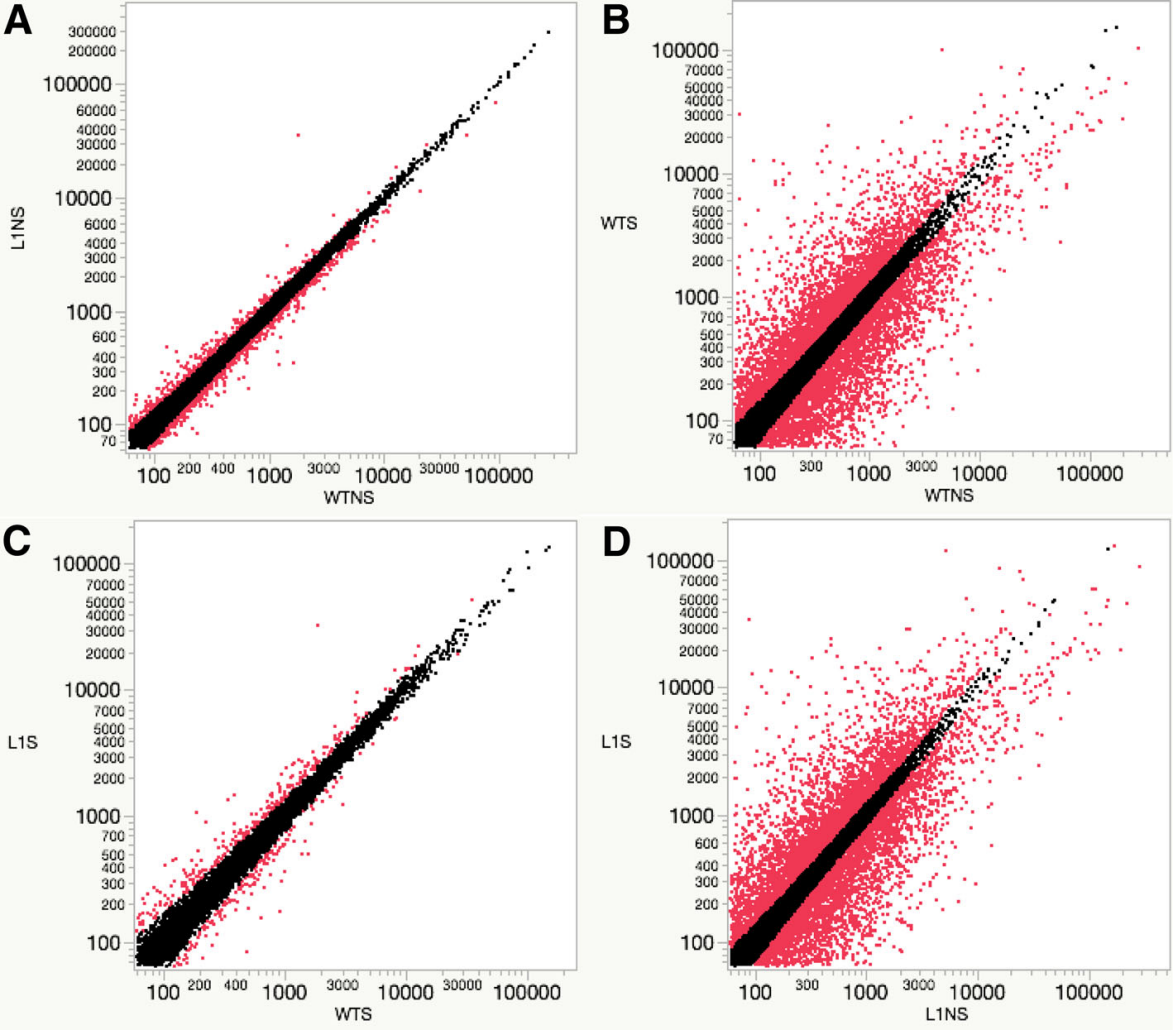
Scatter plot showing significantly differential gene expression (*p* < .05) comparing different rice lines and/or conditions. **a** Comparison of wild type under normal condition (WTNS) and 35S-*OsCam1–1* under normal conditions (L1NS), **b** wild type under normal conditions (WTNS) and wild type under stress conditions (WTS). **c** wild type under stress conditions (WTS) and 35S-*OsCam1–1* under stress conditions (L1S). **d** 35S-*OsCam1–1* under normal conditions (L1NS) and 35S-*OsCam1–1* under stress conditions (L1S). The X and Y axes represent the base mean for the RNA-seq data

To compare the transcriptome profiles of the rice, differential gene expression analysis of the transcriptome data using DESeq [24] was carried out, which provided the number of differentially expressed genes (DEGs) summarized in Table 2. Analysis of the wild type identified 12,184 DEGs (*p* < 0.05) between the transcriptome profile under normal and salt stress conditions (WTNSWTS), in which 5842 and 6342 genes were up-regulated and down-regulated, respectively. For transgenic rice over-expressing *OsCam1–1*, comparisons between normal and salt stress conditions (L1NSL1S) revealed a total of 13,259 DEGs with 6434 and 6825 up-regulated and down-regulated genes, respectively. Furthermore, the transcriptome profiles of the transgenic rice were compared with those of the wild type. Under normal conditions (WTNSL1NS), 2022 DEGs were identified, with 892 and 1130 DEGs expressed at higher or lower levels in the transgenic rice, respectively. Under salt stress, comparisons of transgenic rice with wild type rice (WTSL1S) revealed 1677 DEGs, with 957 and 720 DEGs expressed at higher or lower levels in the transgenic rice, respectively. The scatterplots showed quantitative overview of the four transcriptome profile comparisons (Fig. 1). *OsCam1–1* was found to be highly expressed in transgenic rice under both normal and stress condition, with an average RPKM of 1758.67 and 1644.62, while the average RPKM of wild type under normal and stress conditions was 91.94 and 97.84, respectively. The expression of *OsCam1–1* in the wild type was not induced at 4 h after salt stress (150 mM), in good agreement with a previous study. According to a gene expression study conducted by Chinpongpanich et al. [25], the transcript level of *OsCam1–1* determined by qRT-PCR was highly induced at 1 h after 150 mM NaCl treatment and then sharply decreased after 1 h. This result validated the overexpression of *OsCam1–1* in transgenic rice with an approximately 18-fold change in RPKM compared with wild type. Based on a differential transcriptome analysis, the gene expression levels of those 2022 and 1677 DEGs were thus likely affected by *OsCam1–1* overexpression.

**Table 2:** Differential gene expression analysis results showing the number of significantly differentially expressed genes comparing each rice line and/or condition

Comparison of rice line and/or condition	Total number of differentially expressed genes (p < 0.05)	Number of differentially up-regulated genes (*p* < 0.05)	Number of differentially down-regulated genes (p < 0.05)
WTNSWTS	12,184	5842	6342
L1NSL1S	13,259	6434	6825
WTNSL1NS	2022	892	1130
WTSL1S	1677	957	720

### qRT-PCR verification of the transcriptome data

**Fig. 2:**
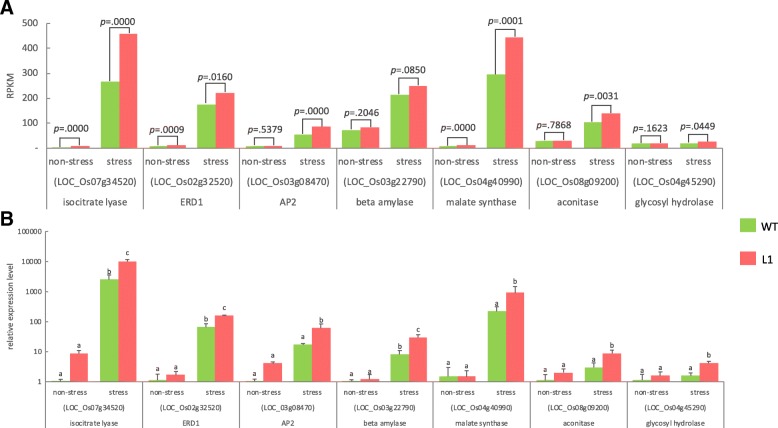
Real time RT-PCR verification of RNA-Seq. **a** RNA-Seq results with *p* values from DESeq analysis, **b** real-time RT-PCR results calculated using the 2^-(ΔΔCT)^ method. Data are shown as the mean + 1 SD, and are derived from four independent biological replicates. For each gene, means with a different letter are significantly different (p < 0.05)

To verify the reliability of the transcriptome data, nine salt-responsive genes, β-amylase (LOC_Os03g22790), isocitrate lyase (LOC_Os07g34520), malate synthase (LOC_Os04g40990), aconitase (LOC_Os08g09200), glycosyl hydrolase (LOC_Os04g45290), ERD1 (LOC_Os02g32520), AP2 (LOC_Os03g08470), isocitrate dehydrogenase (LOC_Os05g49760) and pyruvate decarboxylase (LOC_Os03g18220), were selected for qRT-PCR. Figure 2 shows the qRT-PCR results for seven genes, which agreed well with the transcriptome data. Compared with wild type, they all exhibited higher levels in transgenic rice, demonstrating a statistically significant difference under salt stress. In contrast, the expression of the other two genes examined by qRT-PCR did not agree well with the transcriptome data (data not shown), potentially because of their low expression levels.

### Gene ontology enrichment analysis

**Fig. 3:**
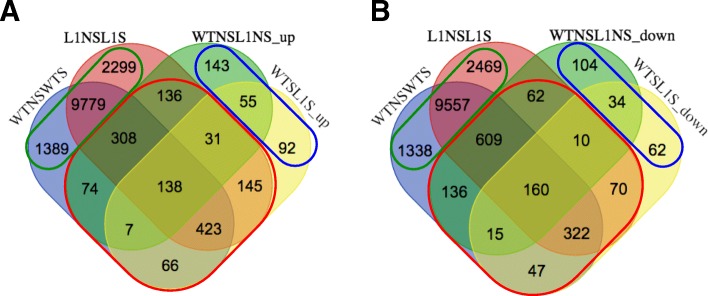
Venn diagram showing (**a**) the number of significantly (p < 0.05) HT salt-responsive DEGs (red circle), HT DEGs (blue circle) and salt-responsive DEGs (green circle), while (**b**) shows the number of LT salt-responsive DEGs (red circle), LT DEGs (blue circle) and salt-responsive DEGs (green circle)

To categorize the DEGs, Venn diagrams were constructed from the four comparisons. The first Venn diagram was constructed from the salt-responsive DEGs from either wild type (WTNSWTS, blue colored circle) or transgenic rice (L1NSL1S, red colored circle) and the DEGs that were expressed at higher levels in the transgenic rice either under normal (WTNSL1NS_up, green colored circle) or salt stress (WTSL1S_up, yellow colored circle) conditions (Fig. 3a). In contrast, the second diagram was constructed from the salt-responsive DEGs and the DEGs that were expressed at lower levels in transgenic rice either under normal (WTNSL1NS_down, green colored circle) or salt stress (WTSL1S_down, yellow colored circle) conditions (Fig. 3b). According to the Venn diagrams, we identified 1328 salt-responsive DEGs with higher expression levels in transgenic rice, which will be referred to as HT salt-responsive DEGs (Fig. 3a, red line circle), and 1431 salt-responsive DEGs with lower expression levels in transgenic rice, which will be referred to as LT salt-responsive DEGs (Fig. 3b, red line circle). For those with unaffected expression levels by salt stress, 290 genes or 200 genes showed higher (Fig. 3a, blue line circle) or lower (Fig. 3b, blue line circle) expression levels in transgenic rice, which will be referred to as HT DEGs or LT DEGs, respectively. In both Venn diagrams, the largest of number DEGs with expression levels that did not differ between transgenic rice and wild type (green circles) were salt-responsive DEGs.

**Fig. 4:**
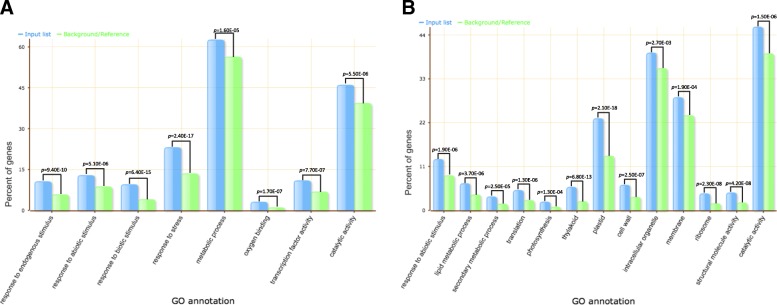
GO enrichment analysis results of (**a**) the salt-responsive DEGs with higher expression levels in transgenic rice and (**b**) the salt-responsive DEGs with lower expression levels in transgenic rice

Gene enrichment analysis was performed using those 1328 HT salt-responsive DEGs. The results showed that, in terms of biological process, the terms of response to endogenous stimulus (GO:0009719), response to abiotic stimulus (GO:0009628), response to biotic stimulus (GO:0009607), response to stress (GO:0006950) and metabolic process (GO:0008152), were enriched, while for the term of molecular function, the terms of oxygen binding (GO:0019825), transcription factor activity (GO:0003700) and catalytic activity (GO:0003824) were overrepresented (Fig. 4a). For those 1431 LT salt-responsive DEGs, in terms of biological process, the terms of response to abiotic stimulus (GO:0009628), lipid metabolic process (GO:0006629), secondary metabolic process (GO:0019748), translation (GO:0006412), and photosynthesis (GO:0015979) were enriched, while in terms of the cellular compartment, the enriched terms included the thylakoid (GO:0009579), plastid (GO:0009536), cell wall (GO:0005618), intracellular organelles (GO:0043229), membrane (GO:0016020) and ribosome (GO:0005840), and in terms of molecular function, those of structural molecule activity (GO:0005198) and catalytic activity (GO:0003824) were overrepresented (Fig. 4b). Based on these results, we observed that the set of genes involving photosynthetic process were uniquely allocated in the LT salt-responsive DEGs, while genes involving response to stimuli and metabolic process were distributed in both HT and LT salt-responsive DEGs.

### Functional identification of OsCam1–1 regulated DEGs

Table 3 summarizes the number of *OsCam1–1*-regulated DEGs in each functional category according to GO terms. For the categories of HT and LT DEGs with expression levels that remained unchanged under salt stress, a small number of genes with diverse functions were found, which were involved in RNA regulation, protein metabolism, signaling, development, transport, hormone, and stress. However, the high-fold-change-DEGs were mainly identified as unknown proteins (see Additional file 1). Nonetheless, some known genes were annotated in the genome database including ankyrin repeat containing protein (LOC_Os01g09384), hexose carrier protein (LOC_Os11g38160), ATPase/hydrogen-translocating pyrophosphatase (LOC_Os01g23580), UDP glucosyltransferase (LOC_Os01g49240), C3HC4-type RING finger (LOC_Os10g32760), cleavage and polyadenylation specificity factor 100 kDa subunit (LOC_Os02g06940).

**Table 3:** Number of *OsCam1–1*-regulated DEGs in each functional category according to GO terms

GO Term	Number of				
	HT salt-responsive DEG	LT salt-responsive DEG	HT DEG	LT DEG	salt responsive DEG
photosynthesis	2	31	0	1	111
cell wall	26	52	0	2	178
lipid metabolism	30	55	1	3	233
N metabolism	4	2	0	0	15
amino acid metabolism	17	21	2	4	155
S assimilation	2	1	0	0	3
metal handling	5	6	2	1	31
secondary metabolism	35	50	9	3	185
hormone	51	27	13	6	227
co-factor and vitamin metabolism	2	5	0	1	35
tetrapyrrole synthesis	0	13	1	0	19
major CHO metabolism	7	7	2	0	61
stress	66	54	14	7	420
redox	6	16	1	2	116
polyamine metabolism	2	0	0	0	8
nucleotide metabolism	8	9	0	0	82
biodegradation of xenobiotics	2	1	0	0	44
C1-metabolism	0	3	0	1	14
miscellaneous	138	145	22	15	750
RNA regulation	126	113	26	15	1318
DNA synthesis	9	25	2	8	224
protein metabolism	130	164	28	17	1575
minor CHO metabolism	11	6	1	2	65
signaling	71	53	18	10	656
cell division	13	48	8	5	329
development	53	36	10	6	330
transport	68	67	11	6	576
not assigned	430	410	118	85	4151
glycolysis	2	0	0	0	29
fermentation	2	5	0	0	11
gluconeogenesis	3	1	0	0	7
OPP	2	1	0	0	15
TCA	2	3	0	0	52
electron transport chain	3	1	1	0	50
micro RNA	0	0	0	0	1
Total	1328	1431	290	200	12,076

**Fig. 5:**
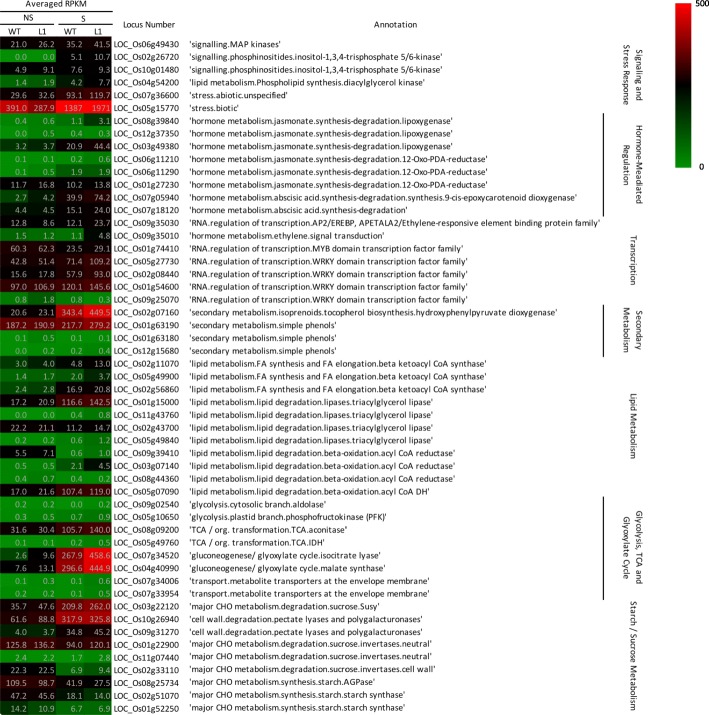
Salt-responsive DEGs that encode potential downstream components of OsCaM1 in salt stress response. Expression levels of each gene by RPKM from wild type and transgenic rice under normal and salt stress conditions were presented as heat map

Overall, under salt stress, 1434 salt-responsive genes exhibited different expression levels between the wild type and transgenic rice. Figure 5 shows salt-responsive DEGs that encode potential downstream components of OsCaM1 in salt stress response. These DEGs are involved in several major cellular processes, including signaling and stress responses, hormone-mediate regulation, transcription, secondary metabolism, lipid metabolism, glycolysis, TCA cycle, glyoxylate cycle, photosynthesis, and carbohydrate metabolism. In signaling, the HT salt-responsive DEGs include LOC_Os06g49430, which encodes BWMK1, a rice MAP kinase; LOC_Os02g26720 and LOC_Os10g01480, which encode inositol 1,3,4-trisphosphate 5/6-kinase (IPTK); and LOC_Os04g54200, which encodes diacylglycerol kinase (DGK). Involved in stress response, the transcriptome results showed that the expression of 46 biotic and 19 abiotic stress DEGs was allocated in the HT salt-responsive DEG category (see Additional file 1). These include a universal stress protein (USP) (LOC_Os07g36600), and a xylanase inhibitor protein gene (*OsXIP2*) (LOC_Os05g15770). For those involved in hormone-mediated regulation, we have identified three lipoxygenase (*LOX*) genes (LOC_Os08g39840, LOC_Os12g37350, and LOC_Os03g49380) and three 12-oxo-PDA-reductase (*OPR*) genes (e.g. LOC_Os06g11210, LOC_Os06g11290, and LOC_Os01g27230), which encode enzymes in the jasmonate (JA) biosynthesis pathway. In addition, the genes encoding key enzymes in the ABA biosynthesis pathway, 9-cis-epoxycarotenoid dioxygenase (NCED) (LOC_Os07g05940) and abscisic aldehyde oxidase (AAO) (LOC_Os07g18120), were identified as HT salt-responsive DEGs, which were up-regulated approximately 1.9-fold and 1.6-fold, respectively in transgenic rice compared with wild type rice under salt stress.

According to the transcriptome results, thirteen APETALA2/ethylene-responsive element binding protein (AP2/EREBP) genes were identified as HT salt-responsive DEGs (e.g. LOC_Os09g35030, LOC_Os09g35010), while five AP2/EREBP genes were identified as LT salt-responsive DEGs (see Additional file 1). In addition, eight MYB genes were found allocated in the category of HT salt-responsive DEGs (e.g. LOC_Os01g74410) and 16 HT salt-responsive DEGs were WRKY, which is a large TF family that responds to plant stress (e.g. LOC_Os05g27730, LOC_Os02g08440, LOC_Os01g54600, LOC_Os09g25070). In secondary metabolism, 35 DEGs were identified as HT salt-responsive genes (see Additional file 1) such as a hydroxyphenylpyruvate dioxygenase (HPPD) gene (LOC_Os02g07160) and five laccase genes (e.g. LOC_Os12g15680, LOC_Os01g63180, LOC_Os01g63190).

Functions of several HT salt-responsive DEGs involve in the energy metabolism. These included 30 DEGs in lipid metabolism (see Additional file 1) with examples including three 3-ketoacyl-CoA synthase genes (LOC_Os02g11070, LOC_Os05g49900 and LOC_Os02g56860), four class III lipase genes (LOC_Os01g15000, LOC_Os11g43760, LOC_Os02g43700 and LOC_Os05g49840), and four genes encoding enzymes involving beta oxidation (LOC_Os09g39410, LOC_Os03g07140, LOC_Os08g44360 and LOC_Os05g07090). Several HT salt-responsive DEGs are also involved in carbohydrate metabolism including a fructose bisphosphate aldolase (FBP) gene (LOC_Os09g02540) and a phosphofructokinase (PFK) gene (LOC_Os05g10650) in the glycolysis pathway; and an aconitase gene (LOC_Os08g09200) and an isocitrate dehydrogenase (IDH) gene (LOC_Os05g49760) in the TCA cycle. In addition, two genes encoding key enzymes in the glyoxylate cycle shuttling the TCA cycle pathway, isocitrate lyase (ICL) (LOC_Os07g34520) and malate synthase (MLS) (LOC_Os04g40990) were up-regulated approximately 1.7-fold and 1.5-fold, respectively in transgenic rice compared with wild type rice under salt stress. Additionally, the DEGs included two glucose-6-phosphate transporter genes, LOC_Os07g34006 and LOC_Os07g33954, both with higher expression levels in transgenic rice under salt stress.

Finally, seven DEGs involved in sucrose and starch metabolism were allocated in the category of HT salt-responsive DEGs. Among these DEGs, sucrose synthase (LOC_Os03g22120) was up-regulated in both wild type or transgenic rice under salt stress, with greater up-regulation in transgenic rice. The transcriptome data also revealed three invertase genes (LOC_Os01g22900, LOC_Os11g07440, LOC_Os02g33110) and seven cell wall degradation DEGs, which were expressed at higher levels in transgenic rice (see Additional file 1) including two DEGs encoding polygalacturonase (LOC_Os10g26940 and LOC_Os09g31270).

**Fig. 6:**
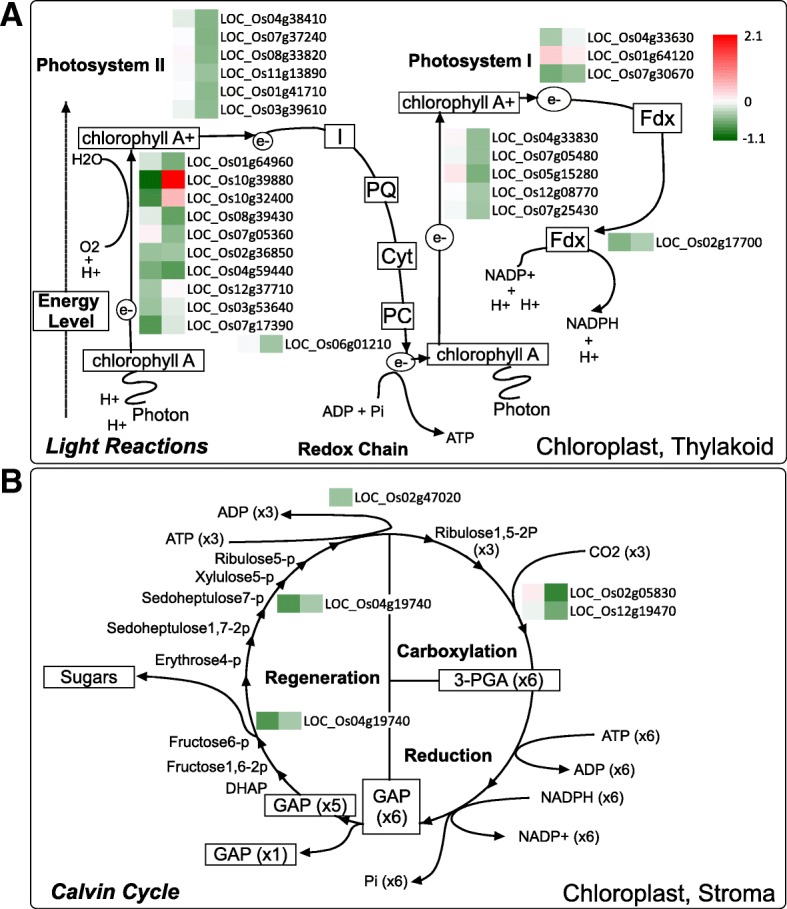
Photosynthetic pathway showing the expression level and role(s) of the genes in the light reaction (**a**) and Calvin cycle. **b** The left box shows the log2-fold change in comparisons of WT and transgenic plants under normal conditions, and the right box represents the log2-fold change under salt stress conditions

**Fig. 7:**
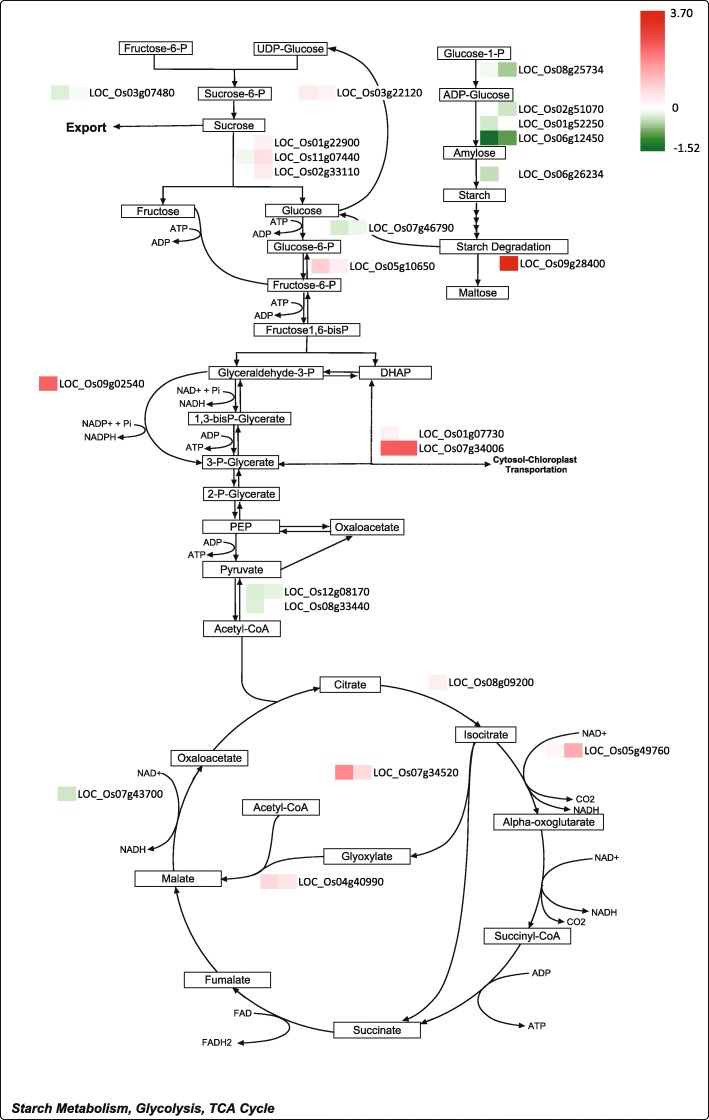
Carbohydrate and energy metabolism pathway consisting of sucrose-starch metabolism, glycolysis and the TCA cycle show the gene expression level and function in metabolism. The left box shows the log2-fold change of comparisons of WT and the transgenic plants under normal conditions, and the right box represents the log2-fold change under salt stress conditions

When these salt-responsive DEGs were mapped onto metabolic pathways, several genes with consistent changes in their expression levels within certain pathways were observed, including the light reactions and Calvin cycle of the photosynthetic process, sucrose and starch metabolism, and central energy pathways. In Fig. 6 and Fig. 7, gene expression ratios between the transgenic rice and the wild type rice both under normal and salt stress conditions are presented for each corresponding step of these pathways. Overall, expression levels of 31 out of 33 DEGs in the photosynthetic process (e.g., chlorophyll a/b binding protein, protein subunit in photosystem I and II, ferredoxin, plastoquinone dehydrogenase complex, ribulose-bisphosphate carboxylase, which were repressed by salt stress) were lower in the transgenic rice overexpressing *OsCam1–1* (Fig. 6) (see Additional file 2). In Fig. 7, the expression levels of several genes involved in sucrose degradation (e.g., LOC_Os03g22120 encoding sucrose synthase, which was highly induced by salt stress; LOC_Os01g22900 and LOC_Os02g33110 encoding invertase, which were repressed by salt stress) were higher in transgenic rice, especially under salt stress, while those of genes in the starch biosynthetic pathway (e.g., LOC_Os08g25734 encoding glucose-1-phosphate adenylyltransferase; LOC_Os02g51070 and Os01g52250 encoding starch synthase, which were repressed by salt stress) were lower in transgenic rice. In addition, genes involved in glycolysis and the TCA cycle (e.g., LOC_Os05g10650 encoding phosphofructokinase; and LOC_Os05g49760 encoding isocitrate dehydrogenase, which were induced by salt stress) were expressed at higher levels in transgenic rice. Remarkably, three genes in the glyoxylate cycle: LOC_Os08g09200, LOC_Os07g34520, and LOC_Os04g40990 encoding aconitase, isocitrate lyase, and malate synthase, respectively, which were all highly induced by salt stress, were expressed at higher levels in transgenic rice both under normal and salt stress conditions (Fig. 7).

### Rice overexpressing OsCam1–1 exhibited higher sucrose and starch contents under salt stress

**Fig. 8:**
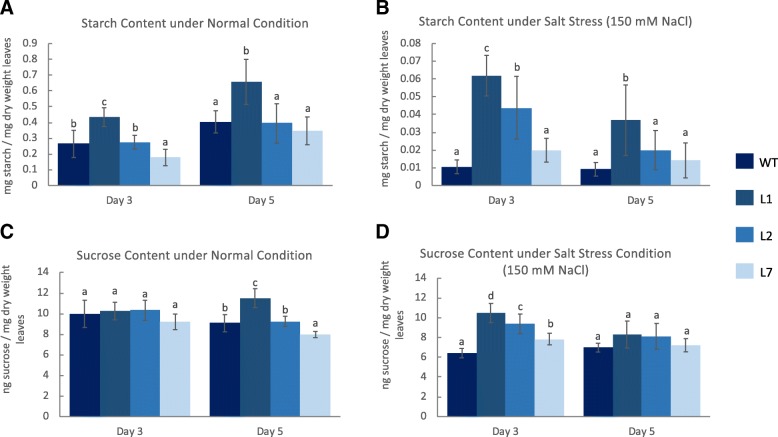
Starch and sucrose contents in the three lines of transgenic rice overexpressing *OsCam1–1* (L1, L2, L7) comparing wild type (WT) at days 3 and 5 exposed to 150 mM NaCl salt stress treatment from five independent biological replicates. **a** starch content under normal conditions, **b** starch content under salt stress conditions, **c** sucrose content under normal conditions, and (**d**) sucrose content under salt stress conditions

In our previous report [17], *OsCam1–1*-overexpressing lines showed a significantly higher relative growth rate than wild type when grown under salt stress. Based on the genes identified herein, among which several were involved in central energy pathways, sucrose and starch levels were determined in the three independent lines (L1, L2, L7) under normal and salt stress (150 mM NaCl) conditions at day 3 and 5 after treatment. Salt stress led to a significant reduction of the starch level and slightly decreased sucrose levels in both wild type and transgenic rice lines. Noticeably, at day 3, the transgenic lines could maintain the sucrose and starch levels better than the wild type under salt stress conditions. At day 5, the trends observed for sucrose and starch levels in transgenic rice under salt stress conditions were similar to those in wild type (Fig. 8).

**Fig. 9:**
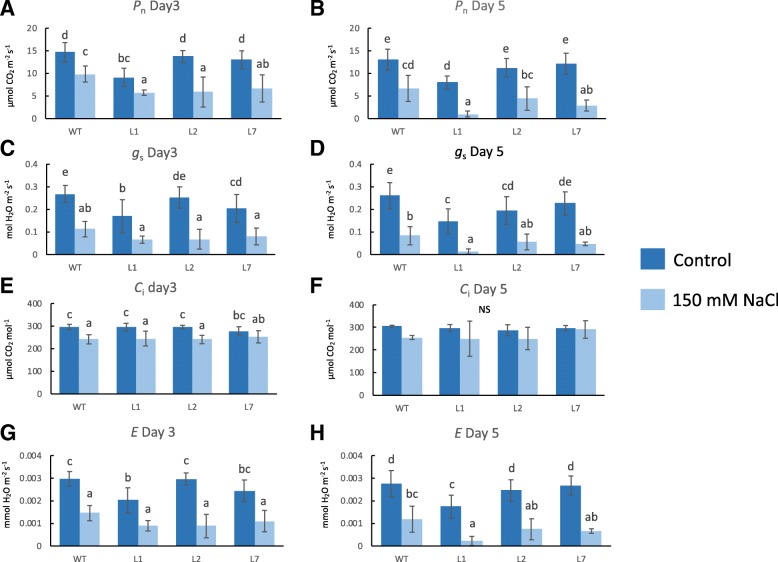
Gas exchange measurements in the leaves of three lines of transgenic rice overexpressing *OsCam1–1* (L1, L2, L7) comparing wild type (WT) of 150 mM NaCl salt stress treatment from five independent biological replicates. (**a**) the photosynthesis rate (*P*_n_) at day 3 and (**b**) day 5, (**c**) stomatal conductance (*g*_s_) at day 3 and (**d**) day 5, (**e**) intercellular carbon dioxide (*C*_i_) at day 3 and (**f**) day 5, and (**g**) transpiration rate (*E*) at day 3 and (**h**) day 5 of salt stress treatment

In addition, the photosynthesis rate (*P*_n_), stomatal conductance (*g*_s_), intercellular carbon dioxide (*C*_i_) and transpiration rate (*E*) were examined in the transgenic rice over-expressing *OsCam1–1*. Under salt stress, *P*_n_, *g*_s_ and *E* decreased at both day 3 and day 5, while *C*_i_ decreased slightly at day 3 of treatment. Interestingly, transgenic rice had slightly lower *P*_n_ values than wild type rice at both day 3 and day 5 and tended to have lower *g*_s_ and *E* values at day 5 of salt stress treatment. In contrast, the *C*_i_ measurements did not reveal significant difference between the transgenic and wild type (Fig. 9). For FV′/FM′, which reflects the maximum efficiency of photosystem II [26], no change was observed under the given salt stress conditions, and the transgenic rice did not exhibit difference either under normal or salt stress conditions compared with the wild type (see Additional file 3).

In a previous study, the northern blot results showed the highest expression levels of *OsCam1–1* in transgenic rice line L1 among the three transgenic rice lines [17]. Under both normal and salt stress conditions, the sucrose and starch content correlated with the expression level of *OsCam1–1* in those transgenic rice lines.

### Identification of OsCaM1-interacting proteins

CaM does not possess functional domains other than EF hand motifs, so it functions by binding to and altering the activities of various interacting proteins. To understand how CaM1 mediates Ca^2+^-signal responses, its specific interacting proteins were identified using a cDNA expression library with ^35^S-labeled rOsCaM1 protein as the probe. The purity of the prepared ^35^S-labeled rOsCaM1 protein was examined by SDS-PAGE (see Additional file 4**:** Figure S3A). To test its specificity, PVDF membrane spotted with various amounts of CaMKII peptide [27], calcineurin [28], and BSA was incubated with the probe. The autoradiograph (see Additional file 4: Figure S3B) showed that the probe only interacted with CaMKII peptide and calcineurin but not BSA, and the intensity of the signal on the X-ray film was dose-dependent. The results indicated that the ^35^S-labeled rOsCaM1 protein could specifically bind to well-known target proteins in the presence of Ca^2+^.

After screening the cDNA library, the purified clones from the tertiary screening were titered before performing single-clone excision. As a result, 10 distinct positive cDNA clones were obtained. All unique pBluescript SK(−) plasmids obtained from the single-clone excision were sequenced to determine the cloned cDNA insert sequences. The resulting sequences were BLAST-searched against the Rice Genome Annotation Project (MSU-RGAP) and the Rice Annotation Project (RAP) databases [29]. The functions of 8 OsCaM1 targets were identified (Table 4), which were diverse and potentially involved in various cellular processes, including metabolism, transcription, movement of organelles and vesicles, membrane transport, and signal transduction. Four known CaM-binding proteins previously identified in other plants were obtained from this screening, which included a cyclic nucleotide-gated ion channel [30] (LOC_Os06g33570), a glutamate decarboxylase [31] (LOC_Os03g51080), a CaM-binding transcription activator (CAMTA) [32] (LOC_Os04g31900), and a kinesin motor domain-containing protein [33] (LOC_Os04g57140). The six identified putative novel CaM1-binding proteins comprised a transferase family protein (LOC_Os02g39850), a response regulator receiver domain-containing protein (LOC_Os09g36220), a lipin (LOC_Os05g38710), a myosin heavy chain-containing protein (LOC_Os12g17310), and two proteins with unknown function, LOC_Os08g34060 and LOC_Os02g13060. Interaction of eight putative target proteins (Table 4) with OsCaM1 was confirmed by protein blot analysis (see Additional file 5).

**Table 4:** OsCaM1 target gene list obtained by cDNA expression library screening

Locus	Gene Annotation	Chromosome	ORF (bp)	Protein (aa residues)	Protein Blot Confirmation
LOC_Os06g33570	cyclic nucleotide-gated ion channel	6	2085	694	+
LOC_Os03g51080	glutamate decarboxylase	3	1533	510	+
LOC_Os04g31900	calmodulin-binding transcription activator	4	3012	1003	+
LOC_Os04g57140	kinesin motor domain containing protein	4	3588	1195	+
LOC_Os02g39850	hydroxyanthranilate hydroxycinnamoyltransferase	2	1329	442	+
LOC_Os09g36220	response regulator receiver domain-containing protein	9	1872	623	–
LOC_Os05g38710	lipin, N-terminal conserved region family protein	5	2655	884	+
LOC_Os12g17310	myosin heavy chain-containing protein	12	1944	647	+
LOC_Os08g34060	DUF1336 domain containing protein, expressed	8	2292	763	^a^ND
LOC_Os02g13060	expressed protein	2	699	232	+

^a^ND represents not determine

### Statistical verification of OsCam1–1 affected DEGs and OsCaM1 targets

Overall, by RNA Seq, 3249 genes were found to be differentially expressed between *OsCam1–1*-overexpressing rice and wild type. To confirm the validity of this gene list, a statistical approach was employed using Fisher’s exact test to determine the statistical confidence of the data as being true. Of the 55,986 rice genes according to the MSU7 rice genome database (http://rice.plantbiology.msu.edu/analyses_facts.shtml) [23], 60 rice genes were co-expressed with *OsCam1–1* by co-expression analysis using the web-based tool STRING (https://string-db.org/) [34], which were used as a reference list of known *OsCam1–1*-affected genes (see Additional file 6). Within this supposed known gene set, 30 genes were found in the list of 3249 *OsCam1–1*-affected genes based on the RNA-Seq results (see Additional file 7). By Fisher’s exact test, genes in the known gene set were significantly over-presented in our list of 3249 *OsCam1–1*-affected genes with a calculated *p*-value of 1.52 × 10^− 21^.

Similarly, to confirm the validity of the ten putative OsCaM1 targets identified in this study, 60 rice homologs of known Arabidopsis CaM target proteins [35] were identified and used as a reference list of known OsCaM1 targets in rice (**see** Additional file 8). Within this supposed known protein set, 4 proteins were found in the present study list of 10 OsCaM targets identified by cDNA expression library screening. By Fisher’s exact test, OsCaM1 targets in the known protein set were significantly over-represented in our list of 10 putative OsCaM1 target proteins with a calculated p-value of 2.49 × 10^− 10^.

## Discussion

Based on the transcriptomics analysis, *OsCam1–1* overexpression affects genes in several cellular processes, potentially contributing to rice salt tolerance. As the expression level of *OsCam1–1* in transgenic rice was much higher than that in wild type, even under normal conditions, numerous DEGs exhibited altered expression levels in the transgenic rice (WTNSL1NS, green colored circles), suggesting that their functions likely confer advantages to plants in coping with future salt stress. Approximately 80% of these genes were salt-responsive, indicating a likely effect on the salt stress response of *OsCam1–1* overexpression. Under salt stress, approximately 18.4% of the salt-responsive genes differentially expressed between the transgenic and wild type rice (Table 3), which are further discussed below in detail. However, it should be noted that DEGs with expression levels that remained unchanged under salt stress, categorized as HT DEGs and LT DEGs, might also contribute to the salt tolerance of transgenic rice. However, the high-fold-change-DEGs were mainly identified as unknown proteins (see Additional file 6). Nonetheless, some known genes, which were annotated in the genome database, were found to be involved in salt stress or at least in abiotic stress responses, including ankyrin repeat containing protein (LOC_Os01g09384), hexose carrier protein (LOC_Os11g38160), ATPase/hydrogen-translocating pyrophosphatase (LOC_Os01g23580), UDP glucosyltransferase (LOC_Os01g49240), C3HC4-type RING finger (LOC_Os10g32760), cleavage and polyadenylation specificity factor 100 kDa subunit (LOC_Os02g06940).

### Signaling and stress responses

The mitogen-activated protein kinase (MAPK/MPK) cascade is a highly conserved central regulator of diverse cellular processes [36]. CaM plays role in the MAPK/MPK cascade by binding to mitogen-activated protein kinase (MPK) and/or mitogen-activated protein kinase phosphatase (MKP) [37, 38]. A rice MAPK, BWMK1 encoded by an HT salt-responsive DEG, could phosphorylate the OsEREBP1 transcription factor for binding to the GCC box element (AGCCGCC), which is a basic component of several pathogenesis-related gene promoters [39]. Inositol 1,3,4-trisphosphate 5/6-kinase (IPTK) encoded by two HT salt-responsive DEGs, phosphorylates inositol 1,3,4-trisphosphate to form inositol 1,3,4,5-tetrakisphosphate and inositol 1,3,4,6, tetrakisphosphate, which are ultimately converted to inositol hexaphosphate (IP6) and play roles in plant growth and development [40]. In rice, the T-DNA mutant of an IPTK gene showed reduced osmolyte accumulation and growth under drought conditions, and some genes involved in osmotic adjustment and reactive oxygen species scavenging were down-regulated. In addition, overexpression of *DSM3* (*OsITPK2*) resulted in a decrease in inositol trisphosphate (IP3), and the phenotypes were similar to the mutant under salt and drought stress conditions. These findings suggested that DSM3 might play a role in fine-tune balancing the inositol phosphate level when plants are exposed to stress or during development [41]. Diacylglycerol kinase (DGK) encoded by an HT salt-responsive DEG, catalyzes the conversion of diacylglycerol (DAG) to phosphatidic acid (PA) [42], and PA plays a role in the stress signaling pathway, including the MAPK/MPK cascade [43]. A report has shown that the expression of *OsBIDK1* encoding rice DGK is induced by benzothiadiazole and fungal infection. Moreover, transgenic tobacco constitutively expressing *OsBIDK1* was more tolerant to plant pathogenic virus and fungi [44]. These findings suggest that several genes in the signaling process might be enhanced by *OsCam1–1* under salt stress.

Interestingly, a universal stress protein (USP) gene was identified as an HT salt-responsive DEG. Evidence has shown that the expression of tomato USP (*spUSP*) is induced by drought, salt, oxidative stress and ABA, and overexpression of *spUSP* improves tomato drought tolerance via interactions with annexin, leading to the accumulation of ABA [45]. In addition, a xylanase inhibitor protein gene (*OsXIP2*), was highly expressed and induced by salt stress and *OsCam1–1* overexpression. A previous report has shown that *OsXIP* can be induced by methyl jasmonate and wounding, so it was suggested that OsXIP may play a role in pathogen defense [46]. As many *OsCam1–1* and/or salt stress affecting DEGs involve both biotic and abiotic stresses, *OsCam1–1* may be a component that mediates the crosstalk of biotic stress and abiotic stress responses.

### Hormone-mediated regulation

Plant hormones play a crucial role in acclimation to abiotic stress and regulate the growth and development and often alter gene expression [47, 48]. Our study revealed that the expression of several genes involved with hormones were changed due to the impact of *OsCam1–1* overexpression under salt stress. Lipoxygenase (*LOX*) encoded by three HT salt-responsive DEGs including homologs of *AtLOX2* and *AtLOX5*, is the enzyme in the early step of the jasmonate (JA) biosynthesis pathway [49]*.* JA plays a role in the physiological response in plants under biotic and abiotic stress [50, 51]. An earlier report has shown that the absence of *AtLOX2* expression results in no change under normal conditions, but JA accumulation induced by wounding is absent and the expression of *vsp*, a wound-JA-induced gene, is also suppressed [52]. In addition, three HT salt-responsive 12-oxo-PDA-reductase (*OPR*) genes identified encode JA precursor-catalyzed enzyme that catalyzes the cis-12-oxophytodienoic acid (OPDA) reduction reaction [53]. These findings suggest that the JA content might be enhanced by the overexpression of *OsCam1–1* under salt stress by enhancing the production of enzymes in the JA biosynthesis pathway. In addition, the expression of *NCED* and *AAO* genes participating in ABA biosynthesis [54] was altered by the influence of *OsCam1–1* overexpression under salt stress. ABA biosynthesis is activated by abiotic stress through Ca^2+^ signaling and the phosphorylation cascade [55]. Our previous report has shown that the expression levels of *NCED* and *AAO*, and ABA content are enhanced in transgenic rice over-expressing *OsCam1–1* under salt stress in comparison to wild type [17].

Collectively, the transcriptome indicates that *OsCam1–1* overexpression likely has effects across biotic and abiotic stresses via plant hormonal regulation through JA and ABA. It has been suggested that biotic-abiotic stress crosstalk may occur via the MAPK/MPK cascade to regulate the plant hormone response to stress [56].

### Transcription

Transcription factors (TFs) play roles as master regulators controlling clusters of genes [57] in the plant regulation of the stress response [58]. AP2/EREBP encoded by several HT salt-responsive DEGs, is in a large gene family of TFs that function in plant growth, primary and secondary metabolism, and response to hormones and environmental stimuli [59, 60]. Two AP2/EREBP DEGs were identified as OsDREB1A and OsDREB1B [61], and a previous report has shown that OsDREB1A-overexpressing transgenic Arabidopsis exhibit induced expression of target stress-inducible genes of Arabidopsis DREB1A and increased tolerance to drought, high salt and freezing stress, as compared with wild type [62].

MYB, which was found encoded by several HT salt-responsive DEGs, is an important gene family of TFs, and several Arabidopsis MYB genes respond to hormone(s) or stress [63]. A previous report has shown that overexpression of *OsMYB48–1*, which is a member of those DEGs, resulted in enhanced salt and drought tolerance in rice. Furthermore, OsMYB48–1 also controlled ABA biosynthesis by regulating the expression of *OsNCED4* and *OsNCED5* in response to drought stress [64].

WRKY is a large TF family that responds to plant stress by regulating the plant hormone signal transduction pathway and is also involved in the biosynthesis of carbohydrate and secondary metabolites, senescence, and development [65]. According to several reports, WRKY genes identified here as HT salt-responsive DEGs are involved in the biotic stress response. The evidence shows that OsWRKY53 can bind to mitogen-activated protein kinases, OsMPK3 and OsMPK6, and inhibit their activity, resulting in a reduction of JA, jasmonoyl-isoleucine and ethylene production and causing a suppression of herbivore defense ability [66]. The expression of OsWRKY71 was induced by salicylic acid (SA), JA, and 1-aminocyclo-propane-1-carboxylic acid (ACC). Overexpression of OsWRKY71 affected the induction of *OsNPR1* and *OsPR1b* expression, which are defense signaling genes, resulting in an enhancement of bacterial plant pathogen resistance [67]. WRKY13 has been shown to regulate crosstalk between abiotic and biotic stress by suppressing the SNAC1 and WRKY45–1 genes, which are involved in drought and bacterial infection, by binding to W-like-type cis-elements on their gene promoters [68]. OsWRKY62, which was down-regulated by effect of *OsCam1–1* overexpression and salt stress, was found in two splicing forms, short and full-length forms. Overexpression of the full-length form of OsWRKY62 resulted in the suppression of blast fungus resistance. In contrast, the knockout *Oswrky62* line showed an enhanced defense-related gene expression level and accumulation of phytoalexins [69].

Based on the transcriptome profiles, *OsCam1–1* overexpression clearly affected the expression of transcription factors that are well-known to regulate both biotic and abiotic stress responses. Therefore, *OsCam1–1* likely functions through the activity of these transcription factors in mediating biotic-abiotic crosstalk regulation via diverse mechanisms. According to our transcriptomics data analysis, plant hormones might mediate the regulation of these TFs, leading to the downstream acclimated phenotypes in response to diverse stresses.

### Secondary metabolism

Secondary metabolites play important roles in acclimating the plant to the environment and stress conditions [70]. A hydroxyphenylpyruvate dioxygenase (HPPD), which participates in the first committed reaction in the vitamin E biosynthesis pathway [71], was highly expressed and enhanced by the effect of either *OsCam1–1* overexpression or salt stress. Previous evidence has shown that the expression of *HPPD* responded to oxidative stress in barley leaf because it was induced by senescence, methyl jasmonate, ethylene, hydrogen peroxide and herbicide; paraquat and 3-(3,4-dichlorophenyl)-1,1-dimethylurea [72]. Furthermore, a report on the expression of two rice laccase (*LAC*) genes (LOC_Os01g63180 and LOC_Os12g15680), which were identified as HT salt-responsive DEG here, in yeast cells suggested that the laccases played roles in atrazine and isoproturon herbicide detoxification [73]. In Arabidopsis, *atlac1* and *atlac2* mutants exhibited deficient root elongation under polyethylene glycol (PEG) treatment, while the *atlac8* mutant showed early flowering and the *atlac15* mutant showed abnormal seed color. In addition, the evidence revealed that the expression level of *AtLAC2* was enhanced by salt and PEG treatment [74].

### Lipid metabolism

Previous evidence has shown that 3-ketoacyl-CoA synthase encoded by three HT salt-responsive DEGs, plays a role in wax biosynthesis. The *kcs1–1* mutant exhibited reduced wax content, a thin seedling stem and low moisture sensitivity [75]. Another report has shown that the expression of *KCS20* and *KCS2/DAISY*, two other Arabidopsis 3-ketoacyl-CoA synthase genes, is induced by salt, ABA and drought conditions and that these genes play roles in cuticular wax and suberin biosynthesis in root [76]. In addition, previous evidence has shown that Arabidopsis genes encoding class III triacylglycerol lipase encoded by four HT salt-responsive DEGs, are involved in many processes. At4g16070, which is orthologous to one of HT salt-responsive DEGs, was predicted to be a gene involved in stress or the Ca^2+^ signaling pathway. At4g16820 and At4g18550, which are orthologous to other rice DEGs are involved in seed germination, senescence or the stress response. At1g02660, which is orthologous to another rice DEG, is involved in the plant defense response signaling pathway [77]. Finally, an early report showed that acyl-CoA dehydrogenase, which was identified encoded by another salt-responsive DEG, functions in mitochondrial β-oxidation in maze root tip under glucose starvation conditions [78]. Based on these results, the activity of *OsCam1–1* might affect lipid metabolism and possibly be linked to energy metabolism during salt stress.

### Glycolysis, TCA and Glyoxylate cycle

Glycolysis and TCA cycle are essential in the respiratory pathway to generate energy [79]. Earlier comparative proteomic reports comparing salt-sensitive and salt-tolerant rice strains showed that the expression of FBP, which was identified as an HT salt-responsive DEG, was induced by salt stress in a salt-sensitive rice cultivar [80] and in either salt-sensitive or salt-tolerant strains of barley [81], while the activity of PFK encoded by another HT salt-responsive DEG, was increased under NaCl treatment along with that of pyruvate kinase and phosphoenolpyruvate carboxylase, resulting in an increase in respiratory O_2_ uptake and drastic changes in the levels of glycolytic metabolites in *Bruguiera sexangula* cell cultures [82]. The aconitase gene, which encodes the enzyme that isomerizes citrate to isocitrate in the early step of the TCA cycle, and the isocitrate dehydrogenase (IDH) gene, which encodes the enzyme that catalyzes the oxidative decarboxylation of isocitrate in the TCA cycle were also found in the HT salt-responsive DEG category. Previous studies have shown that in addition to its other function as an RNA binding protein, aconitase mediates resistance to oxidative stress in plants [83] and overexpression of maize *IDH* in Arabidopsis enhances salt tolerance in Arabidopsis [84].

In addition, early reports suggested that ICL and MLS, which were identified as HT salt-responsive DEGs here, play a role in converting lipids to sugar using an acetyl unit from β-oxidation to generate the substrate of gluconeogenesis, and this process is important during the post-germination stage in Arabidopsis [85, 86]. Additionally, the HT salt-responsive DEGs included two glucose-6-phosphate transporter genes. A previous study has demonstrated that the transcript level of glucose-6-phosphate/phosphate translocator (GPT2) in Arabidopsis correlates with the sugar level in leaf [87], and another study has suggested that GPT2 functions as a plastid anti-porter transporting glucose-6-phosphate into the plastid to support starch biosynthesis [88]. Recently, a proteomic study has found that cucumber seed germination is enhanced by melatonin under high salt conditions via regulated energy metabolism and the up-regulation of proteins involved in glycolysis, the TCA cycle and the glyoxylate cycle [89]. The transcriptome results herein illustrated possible changes in the cellular respiratory pathway in transgenic rice under salt stress. These lines of evidence suggest that *OsCam1–1* may confer salt tolerance by regulating central energy metabolism.

### Starch and sucrose metabolism

Salt stress inhibited the activity of granule-bound starch synthase (GSSB) and suppressed the expression of *GSSBI* and *GSSBII*, resulting in a decrease in starch content in rice leaf [90]. An earlier report has shown that Pokkali, the standard salt-tolerant rice, shows significantly higher starch concentration under salt stress than KDML 105, which was identified as a salt-sensitive cultivar [91]. The transgenic KDML105 rice examined herein exhibited significant decrease in starch levels, but to a lesser extent than the wild type, while it showed improved maintenance of sucrose levels under salt stress, which probably reflected the higher salt tolerance ability. The transcriptome results revealed that several sucrose and starch degradation genes were up-regulated. An early report revealed that sucrose synthase, which its gene expression level was up-regulated to a higher level in transgenic rice, plays a role in starch synthesis by generating ADP-glucose or UDP-glucose through the cleavage of sucrose, which can be used for starch polymerization. Moreover, the findings showed that sucrose synthase activity correlated with starch and ADP-glucose accumulation in developing barley seed [92] and that transgenic potato plants with a disrupted sucrose synthase gene were defective in starch accumulation [93]. However, the transcriptome results herein showed that lower expression levels of several genes in the starch biosynthetic pathways in transgenic compared with wild type rice. These findings suggested that starch metabolism in higher plant might be regulated by several mechanisms: post-translational modifications such as redox modulation and protein phosphorylation, or allosteric modulation by metabolites, which is related to the metabolic flux [94]. Additionally, three invertase genes, encoding a sucrose-digesting enzyme, were identified as HT salt-responsive DEGs. A double mutant of two isoforms of Arabidopsis neutral invertase genes, *inv1*/*inv2*, has been shown to exhibit severe growth defects, and therefore the authors suggested that cytosolic invertase may play role in supplying sucrose to Arabidopsis non-photosynthetic cells [95].

Together with the altered expression levels of these genes, our results for increased starch and sucrose levels in transgenic rice suggest that starch and sucrose metabolism are likely downstream components that are regulated by *OsCam1–1* under salt stress. The down-regulation of photosynthetic genes due to the impact of *OsCam1–1* and up-regulation of genes involved in lipid metabolism suggests that transgenic rice may balance carbon and energy metabolism under salt stress by obtaining monosaccharide units through the mobilization of lipids, which might be converted to sugar via the glyoxylate cycle and gluconeogenesis, as in previous discussions and/or the cell wall, which is a large carbon reservoir in the cell.

### OsCaM1 targets elucidating OsCaM1 downstream components

Four known CaM-interacting proteins (CIPs) previously identified in other plants were obtained from the rice cDNA expression library screening indicating specific CaM target identification. Cyclic nucleotide-gated channels (CNGCs), one of the four known CIPs [30], are activated by binding cyclic nucleotide monophosphates, which play roles in ion-homeostasis control, development, and biotic or abiotic stress defense [96]. In Arabidopsis, AtCNGC10 functions in cation uptake in root, and AtCNGC10 antisense Arabidopsis lines are more salt-sensitive than wild type [97]. Another well-known CIP identified herein, glutamate decarboxylase (GAD), is the enzyme that converts L-glutamate to γ-amino butyric acid (GABA), which is involved in amino acid metabolism. CaM binds to GAD and regulates its activity, resulting in a balance of glutamate-GABA metabolism. Transgenic tobacco expressing petunia GAD lacking the CaM-binding domain exhibits a severely abnormal morphology associated with a high level of GABA but low level of glutamate [98].

Calmodulin-binding transcription activators (CAMTAs) are found in several species of multicellular organisms [32]. Herein, an OsCAMTA was confirmed to be a CaM target, even though the transcriptome showed that overexpression of *OsCam1–1* and salt stress did not significantly affect the expression levels of its gene. In Arabidopsis, CAMTA3 regulates a set of biotic stress-responsive genes, and the *camta3* Arabidopsis mutant showed enhanced biotic stress tolerance [99]. A rice kinesin motor domain-containing protein [33] was also confirmed as a CaM target in this study. Some evidence has revealed that kinesin motor domain-containing protein plays a role in cell developmental processes. In late anaphase, the amino-terminal motor kinesin (AtPAKRP1) is accumulated along the microtubule toward the spindle mid-zone and then localized to microtubules near the future cell plate area, suggesting that AtPAKRP1 may play a role in the maintenance or establishment of the phragmoplast microtubule array [100]. Another report has revealed that AtPAKRP2 first exhibits a punctate pattern in late anaphase, and then is concentrated at the division site following the appearance of the phragmoplast microtubule array in the mirror pair. Treatment with brefeldin A, which inhibits protein transportation from the endoplasmic reticulum to the Golgi apparatus, resulted in the alteration of AtPAKRP2 localization, so the authors suggested that AtPAKRP2 functions in Golgi-derived vesicles transportation in the phragmoplast [101].

Here, six CaM-interacting proteins that have not been found in other plant species were identified in rice. In Arabidopsis, hydroxycinnamoyl-coenzyme A shikimate/quinate hydroxycinnamoyltransferase (HCT) (AT5G48930), a homolog of LOC_Os02g39850, which was identified as CIP herein, contains acyltransferase activity capable of catalyzing the conversion *p*-coumaroyl-CoA to caffeoyl-CoA, which plays a role in the lignin biosynthesis pathway. Silencing of this acyltransferase gene in Arabidopsis results in a dwarf phenotype and change in lignin composition [102].

LOC_Os09g36220 was identified as *OsPRR95*, a pseudo response regulator, which takes part in circadian systems by binding a core oscillator to define rhythm to adapt to the daily changing environment [103]. OsPRR95 corresponds to Arabidopsis PRR, AtPRR5 or AtPRR9 [104]. A report has revealed that triple mutant *prr9–11 prr7–10 prr5–10* Arabidopsis exhibit better salt, drought and cold tolerance than wild type, and thus suggested that PRR5, PRR7 and PRR9 are involved in the diurnal cold stress-initiating stress response by mediating the cyclic expression of stress response genes, including *DREB1/CBF* [105]. Additionally, *Mesembryanthemum crystallinum* (ice plant) CSP1, which is a class of pseudo-response regulator-like proteins, co-localizes with calcium-dependent protein kinase (McCDPK1) in the nucleus of NaCl-stressed ice plants, suggesting that it may be regulated by McCDPK1 through reversible phosphorylation [106].

According to the MSU7 database, LOC_Os05g38710, the novel CaM1 target, is annotated as lipin, and the mRNA sequence of LOC_Os05g38710 is annotated as phosphatidate phosphatase (*PAH1*) [107]. A report has demonstrated that the N- and C-terminal regions of mammalian lipin protein share sequence similarity to yeast *PAH1* [108]. Phosphatidate phosphatase is the enzyme that converts phosphatidic acid to diacylglycerol and P_*i*_ [109]. In *Phaseolus vulgaris* cotyledons, phosphatidate phosphatase is stimulated by Ca^2+^ or CaM with Ca^2+^, and a possible role of Ca^2+^-second-messenger in membrane-lipid degradation initiation has been suggested [110]. Therefore, its identification as a CaM-interacting protein herein suggests that Ca^2+^/CaM stimulates phosphatidate phosphatase via direct binding.

**Fig. 10:**
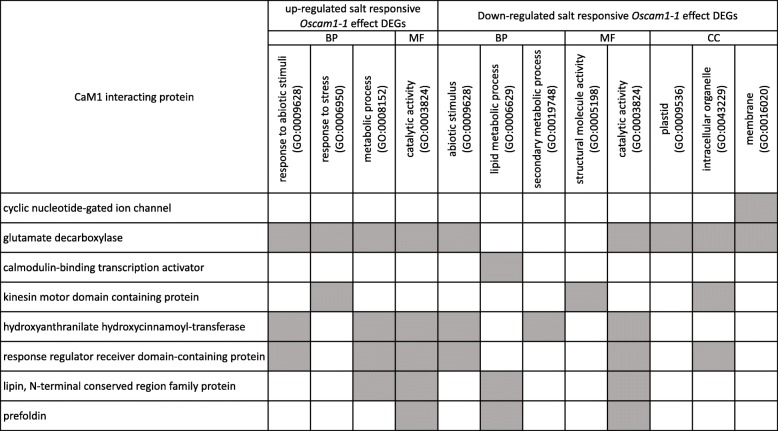
The matched GO term of either up- or down-regulated *OsCam1–1*-effect genes obtaining from RNA-seq versus genes interacting with OsCaM1 targets from the STRING database

By protein functional association analysis of each of these CIPs, the GO terms enriched in each set of resulting associated proteins that matched those from *OsCam1–1* affected salt-responsive DEGs are presented in Fig. 10. Matched GO terms revealed interacting protein candidates that potentially regulate various cellular processes represented by each enriched GO term of the *OsCam1–1* affected salt-responsive DEGs.

## Conclusion

Transcriptome profiling revealed that 18.4% of salt-responsive DEGs are affected by *OsCam1–1* overexpression, which has been previously found to confer salt stress tolerance to transgenic rice [17]. GSEA showed that the DEGs are mainly enriched in the terms of stress response and metabolic process, suggesting that overexpression of *OsCam1–1* confers tolerance by regulating a wide range of processes, including signaling and stress response, hormone-mediated regulation, transcription, secondary metabolism, lipid metabolism, photosynthesis, carbohydrate and energy metabolism. The transcriptome data suggest that CaM action results in an enhancement of metabolic enzymes involved in central energy pathways, which may lead to the mobilization of carbon sources that benefits plant acclimation under salt stress during periods of decreased photosynthesis. The CaM1 target screening results consolidated knowledge that CaM1 downstream components are diverse, involving several cell systems and constituting a complex network of downstream components, as represented by the *OsCam1–1* affected salt-responsive DEGs involved in CaM-mediated salt stress response mechanisms in rice.

## Methods

### Plant materials and stress treatment

The rice *Oryza sativa* L. “Khao Dawk Mali 105” (KDML 105) and transgenic KDML 105 rice over-expressing *OsCam1–1*, generated in a previous work [17], were grown in a growth chamber (Humanlab, South Korea) with Yoshida’s solution [111] for 1 week and then in the greenhouse for 2 weeks using a completely randomized design (CRD) with two biological replicates. Subsequently, the rice was treated with Yoshida’s solution containing 150 mM NaCl. The pooled tissues of leaf blade and leaf sheet were collected, rapidly frozen in liquid nitrogen and stored at − 80 °C for further analyses.

### RNA isolation

The 3-week-old rice was treated with 150 mM NaCl for 4 h, and then the leaves were collected and ground with liquid nitrogen using a chilled mortar and pestle. Subsequently, total RNA was isolated from the tissue using TRI Reagent® (MRC, USA) following the manufacturer’s instruction. After the RNA was precipitated and dissolved in DEPC-treated water, the concentration of RNA was determined using a spectrophotometer (Eppendorf, Germany) based on A_260_/A_280_, and the quality of the RNA was determined by agarose-gel electrophoresis.

### RNA-seq

cDNA library preparation was initiated by isolating mRNA from the prepared total RNA using Dynabeads® (Ambion™, USA), and then first-strand cDNA was synthesized from the mRNA template using Superscript III® reverse transcriptase (Invitrogen™, USA). The mRNA template was eliminated by RNase H (Thermo Fisher Scientific, USA), and second-strand cDNA was synthesized using DNA polymerase I (Thermo Fisher Scientific, USA). The cDNA was fragmented, and the fragments were modified into blunt end fragments using End Repair Enzyme (New England Biolabs, USA). Deoxyadenosines was added to the 3’end of the fragments by Klenow exo- (New England Biolabs, USA), and then the adapters were ligated to the end of the fragments that would later benefit the sample identification process, as each sample was ligated to a different adapter. The ligated fragments were amplified by PCR using Phusion® High-Fidelity DNA Polymerase (New England Biolabs, USA) with adapter primers, and the quality of the PCR products was checked by gel electrophoresis. Every step was followed by purification using AMPure (Beckman Coulter, USA). The cDNA libraries were subjected to single-read sequencing using an Illumina HiSeq 2000 (Illumina, USA).

### Data analysis

The mRNA sequence data obtained from the genome analyzer were demultiplexed and mapped to MSU rice genome version 7 [23] using the computer command sets, Pipeline of Parentally Biased Expression (POPE) [22]. The gene expression data for the wild type and the transgenic rice under normal and salt stress condition were compared using DESeq [24]. Downstream data analyses, Venn diagram construction by the web-based software at http://bioinformatics.psb.ugent.be/webtools/Venn/, and gene enrichment analysis by agriGO [112] were performed. Pathway analysis was conducted to illustrate genes in the pathway using MapMan software [113].

### Real-time RT-PCR

Rice(s) growing, stress treatment and total RNA isolation were repeated with 4 biological replications. The isolated RNA was treated with DNaseI (Thermo Scientific™, USA) and converted to cDNA using the iScript™ cDNA synthesis kit (Bio-Rad, USA). Real-time RT-PCR was performed using SsoFast EvaGreen Supermix (Bio-Rad, USA) with the CFX96™ Real-Time PCR machine (Bio-Rad, USA) with EF-1-α as an internal control. The DNA primers used in this experiment were as follows: EF-1-α (LOC_Os03g08010) F-5′ATGGTTGTGGAGACCTTC3′, R-5′TCACCTTGGCACCGGTTG3′, aconitase (LOC_Os08g09200) F-5′CATCCTCCCATACGTCATCC3′, R-5′TGTCTCCTGCGGCTTTATTT3′, isocitrate lyase (LOC_Os07g34520) F-5′AGAGCAGCAGCCATGTTCTT3′, R-5′CGTGCGTGCTGTAGTTCAGT3′, malate synthase (LOC_Os04g40990) F-5′CGTACAACCTCATCGTGGTG3′, R-5′CGGAGAAGTTACACGGAGAGA3′, AP2 (LOC_Os03g08470) F-5′CTGTGGAGCTTCGACGACTT3′, R-5′ACAAACACAAACACCGCAAT3′, ERD1 (LOC_Os02g32520) F-5′GAGCCACCTGAATGAGAAGG3′, R-5′TTATATGCCCGAACGAATCC3′, glycosyl hydrolase (LOC_Os04g45290) F-5′GCTCAGGTGTGTGTGGTACA3′, R-5′CGACAGCACACATCCTCTGT3′, β-amylase (LOC_Os03g22790) F-5′ATGGATGATGCCCCCTGT3′, R-5′TTGGGGTACACGTCTCATGT3′, isocitrate dehydrogenase (LOC_Os05g49760) F-5′ TCGAGTCTGGGAAGATGACC3′, R-5′AGTGCATCGGATCACATCAA3′ and pyruvate decarboxylase (LOC_Os03g18220) F-5′AGGACGACACCAGCAAAGAG3′, R-5′ GAGGGAATGGACACAAGGAA3′.

### Sucrose and starch determination

The 3-week-old rice grown in a CRD with five replicates was treated with 150 mM NaCl for 3 and 5 days, and then leaves were collected, lyophilized, ground and weighed. Sucrose extraction was performed according to Cowan A.K. et al. [114], and starch was extracted according to Alison and Samuel [115]. The samples for sucrose measurement were then treated with invertase (Sigma, USA) and those for starch measurement with α- and β-amylase (Sigma, USA), followed by incubation at 37 °C for 2 h. The amounts of sucrose and starch were determined based on glucose using the D-Fructose/D-Glucose test kit (Megazyme, Ireland). Potato starch (Sigma, USA) and sucrose (Sigma, USA) were used to construct standard curves.

### Gas exchange and fluorescence measurements

The photosynthesis rate (*P*_n_), stomatal conductance (*g*_s_), intercellular carbon dioxide (*C*_i_), transpiration rate (*E*) and FV′/FM′ of 3-week-old transgenic and wild type rice, which were hydroponically grown under natural light in a CRD with five replicates, were measured using the LI-6400XT portable photosynthesis system (LI-COR®, USA) at day 3 and 5 of 150 mM NaCl treatment. The same parameters were also measured in untreated plants at each time point, and the data were compared.

### Statistical analysis

Data from real-time RT-PCR, sucrose and starch determination, and gas exchange and fluorescence measurements were compared using analysis of variance (ANOVA), and then the means were compared with Duncan’s multiple range test, with significance accepted at the *p* < 0.05 level. Fisher’s exact test was used to verify that genes in the known gene set were significantly over-presented in the list of *OsCam1–1* affected DEGs or OsCaM1 targets, with significance accepted at the p < 0.05 level.

### Preparation of 35S-labeled rOsCaM1 protein

The rOsCaM1 protein [116] was prepared according to Fromm and Chua [117] to be used as a probe for cDNA library expression screening. Production of the recombinant protein in 50 ml cell culture of BL21(DE3) cells harboring pET-21a(+) containing a complete *OsCam1–1* ORF was induced by IPTG. After 15 min of IPTG induction, 1 mCi or Tran ^35^S Label™ (MP Biomedicals, USA) was added to the culture, and the incubation was continued for 4 h. After centrifugation, the pelleted cells were resuspended in 4 ml B-PER reagent containing 1 U/ml DNase I and 20 μg/ml lysozyme. The homogenate was heated in boiling water for 5 min and centrifuged at 15,000 xg for 10 min. The supernatant was collected, and the ^35^S-labeled rOsCaM1 protein was purified by phenyl-sepharose chromatography in the presence of Ca^2+^ according to Liao and Zielinski [118].

### cDNA expression library construction

The cDNA library was constructed by RNA isolation of 100 mM NaCl-treated hydroponically grown 1-week-old KDML105 rice using TRI Reagent® (MRC, USA) and mRNA purification using the Illustra™ Quick Prep Micro mRNA Purification Kit (GE Healthcare, UK). cDNA synthesis and cDNA library construction were carried out using the Uni-ZAP XR vector system (Stratagene, USA). The cDNA was ligated to *XhoI-EcoRI* double-digested Uni-ZAP XR vector, and then the ligated mixture was packaged using Gigapack III packaging extract (Stratagene, USA). The packaging was performed using XL1-Blue MRF’ *E. coli* cells, and the cells were cultured on NZY agar plates overlaid with NZY agar containing IPTG and X-gal for blue-white colony selection. The libraries were propagated in XL1-Blue MRF′ *E. coli,* and the propagated libraries were stored in 0.3% (*v*/v) chloroform at 4 °C.

### cDNA expression library screening

Primary screening was carried out using the propagated library according to Chinpongpanich et al. [119]. The 600-μl aliquots of XL1-Bule MRF2 cell suspension were mixed with the equivalent of 25,000 pfu of the amplified library and incubated at 37 °C for 15 min before plating as top agar with the addition of IPTG onto an LB agar plate. A prewetted nitrocellulose membrane was then placed onto each agar plate, followed by incubation at 37 °C overnight. The membranes were lifted, washed with 1X TBS buffer containing 0.05% Tween-20 (TTBS), and incubated in Blocking Buffer (1X TBS buffer containing 0.05% (v/v) Tween-20, 5 mM CaCl_2_ and 3% (*w*/*v*) skim milk). They were then washed twice in TTBS with 5 mM CaCl_2_ and incubated with ^35^S-labeled rOsCaM1 probe solution for 1–4 h. The membranes were washed twice in TTBS with 5 mM CaCl_2_ and air-dried overnight. Probe binding on the membranes was then detected by autoradiography, and positive plaques were selected for secondary and tertiary screening. The cDNA-containing pBluescript phagemid was excised from the Uni-ZAP XR vector using ExAssist helper phage with the *E. coli* SOLR strain, and the pBluescript phagemid was isolated and then sequenced using M13F and M13R universal primers.

### Protein blot analysis

The *E. coli* strain SOLR cells harboring the cloned cDNA insert-containing pBluescript SK(−) plasmids were cultured and induced by IPTG addition at room temperature overnight. The OsCaM1-binding ability of the positive clones was examined using the ^35^S-labeled rOsCaM1 probe. The duplicate blots were incubated in the probe solution and then separately washed in 1X TTBS containing CaCl_2_ or EDTA. Probe binding on the membrane was detected by autoradiography.

## Additional files

Additional file 1: The DEG lists grouped by HT salt-responsive DEG, LT salt-responsive DEG, HT DEG, LT DEG and salt-responsive DEG. This list contains locus number and annotation of the genes. (XLSX 2043 kb)

Additional file 2: The bar-chart showing expression level (RPKM) of photosynthetic DEGs in the rice(s) under both stress and normal condition. Bar-chart showing expression level of photosynthetic DEGs from RNA-seq data comparing between the transgenic rice overexpressing *OsCam1–1* under stress (150 mM NaCl) condition (L1S), wild type under stress (WTS), transgenic under non-stress (L1NS) and wild type under non-stress (WTNS), and the Y axis represent RPKM. (PDF 171 kb)

Additional file 3: The bar-chart showing FV’/FM’ of the rice(s) under either normal or salt-stress condition. Fluorescence measurement in leaf of the transgenic rice overexpressing *OsCam1–1* (L1, L2, L7) and wild type (WT) under normal and salt stress (150 mM NaCl) condition (A) at day 3 and (B) day 5 of treatment. (PDF 31 kb)

Additional file 4: The reliable testing of the 35S-labeled rOsCaM1 binding for testing accuracy and specificity. Examination of the 35S-labeled rOsCaM1 protein, A) 12% SDS-PAGE of the 35S-labeled rOsCaM1 elutes. Lane M: Protein molecular weight marker. Lanes 1–4: Fractions 1–4 of the 35S-labeled rOsCaM1 elute, respectively, and B) Autoradiograph of the blot spotted with various amounts (as written above each spot) of positive (CaMKII peptide and calcineurin) or negative (BSA) control. (PDF 1805 kb)

Additional file 5: Autoradiographs of far western analysis of 11 putative OsCaM1-binding proteins. Lane M: protein molecular weight markers (negative control); Lane P: calcineurin (positive control); Lane N: crude protein extract from the SOLR cells harboring the pBluescript SK(−) plasmid (negative control); Numbers indicated above the lanes are clone No. assigned when these clones were isolated from the primary screening as following: 2, Cyclic nucleotide-gated ion channel (CNGC); 5, glutamate decarboxylase (GAD); 10, Hydroxyanthranilate hydroxyl cinnamoyltransferase (HHT); 12, CaM-binding transcription activator (CAMTA); 19, Kinesin motor domain- containing protein (KCBP); 21, Kinesin motor domain- containing protein (KCBP); 23, Myosin heavy chain; 24, unknown expressed protein, while 18, 26 and 31 which represent response regulator receiver domain-containing protein (PRR), lipin, N-terminal conserved region family protein, and another clone of Kinesin motor domain- containing protein (KCBP) respectively, were unclear. (PDF 8517 kb)

Additional file 6: The list of *OsCam1–1* co-expressed DEGs obtaining from STRING (Excel file). This list contains locus number and annotation of the genes. (XLSX 17 kb)

Additional file 7: The list of shared genes between *OsCam1–1* co-expressed gene obtaining from STRING and DEGs affected by *OsCam1–1* overexpression from RNA-Seq result. This list contains locus number and annotation of the genes. (XLSX 12 kb)

Additional file 8: The list of rice homologous CaM target genes using known Arabidopsis CaM target gene as references. This list contains locus number and annotation of the genes. (XLSX 23 kb)

## Abbreviations

CaM: Calmodulin
*C*_i_: Intercellular carbon dioxide
CIP: CaM-interacting protein
CRD: Completely randomized design
DEG: Differentially expressed gene
*E*: Transpiration rate
*g*_s_: Stomatal conductance
HT: DEGs whose expression levels were higher in the transgenic rice
KDML 105: Khao Dawk Mali 105
L1: Transgenic KDML 105 rice overexpressing *OsCam1–1* Line 1
L2: Transgenic KDML 105 rice overexpressing *OsCam1–1* Line 2
L7: Transgenic KDML 105 rice overexpressing *OsCam1–1* Line 7
LT: DEGs whose expression levels were lower in the transgenic rice
NS: Non-stress / normal condition
*P*_n_: Photosynthesis Rate
POPE: Pipeline of Parentally Biased Expression
RPKM: Read Per Kilobase of Transcript Per Million Mapped Reads
S: Stress condition
TF: Transcription Factor
WT: Wild Type
WTNS: WT under non-stress
WTS: WT under stress
