# Supplementary figures and images for: Downstream components of the calmodulin signaling pathway in the rice salt stress response revealed by transcriptome profiling and target identification
Source: BMC Plant Biol. 2018 Dec 5;18:335. doi: 10.1186/s12870-018-1538-4 (PMC6282272; doi:10.1186/s12870-018-1538-4)

Figure S1

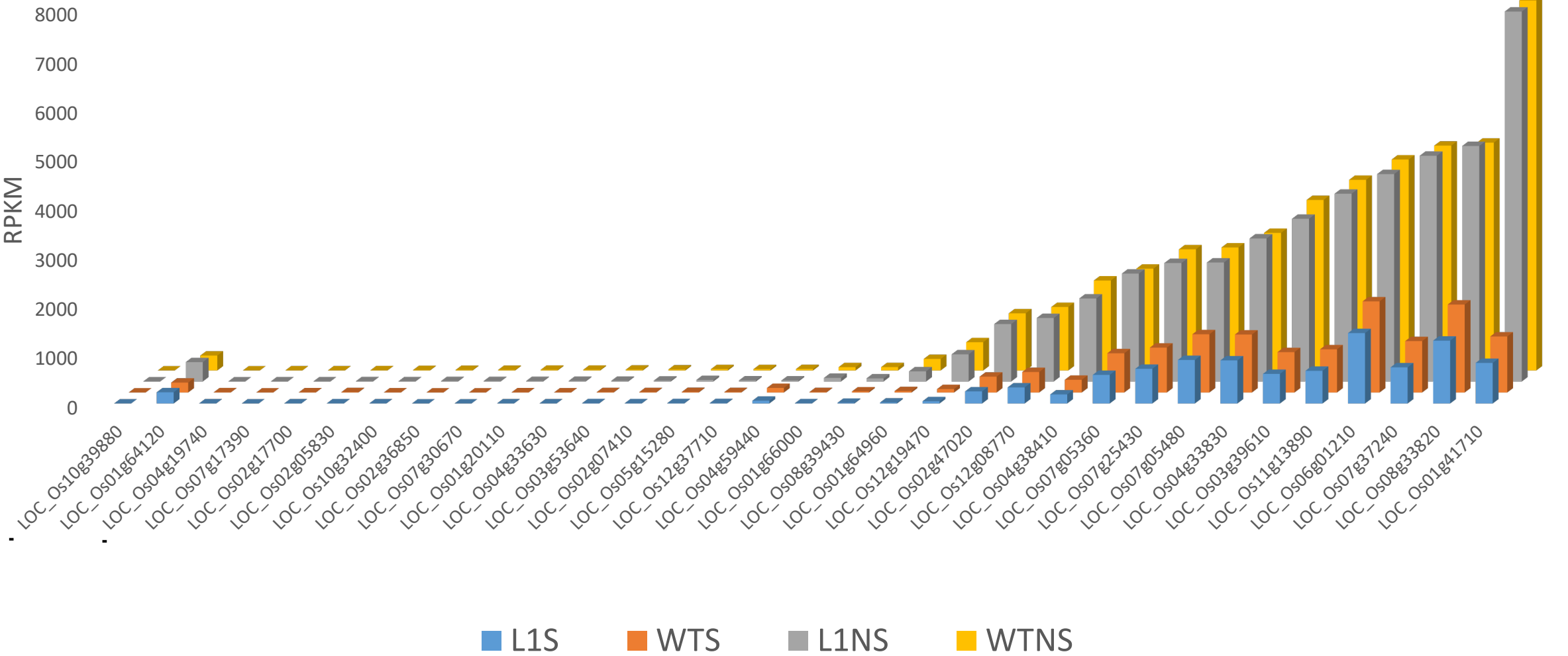

Supplement: Supplementary file 2 — The bar-chart showing expression level (RPKM) of photosynthetic DEGs in the rice(s) under both stress and normal condition. Bar-chart showing expression level of photosynthetic DEGs from RNA-seq data comparing between the transgenic rice overexpressing OsCam1–1 under stress (150 mM NaCl) condition (L1S), wild type under stress (WTS), transgenic under non-stress (L1NS) and wild type under non-stress (WTNS), and the Y axis represent RPKM. (PDF 171 kb) [file 12870_2018_1538_MOESM2_ESM.pdf]

Figure S2

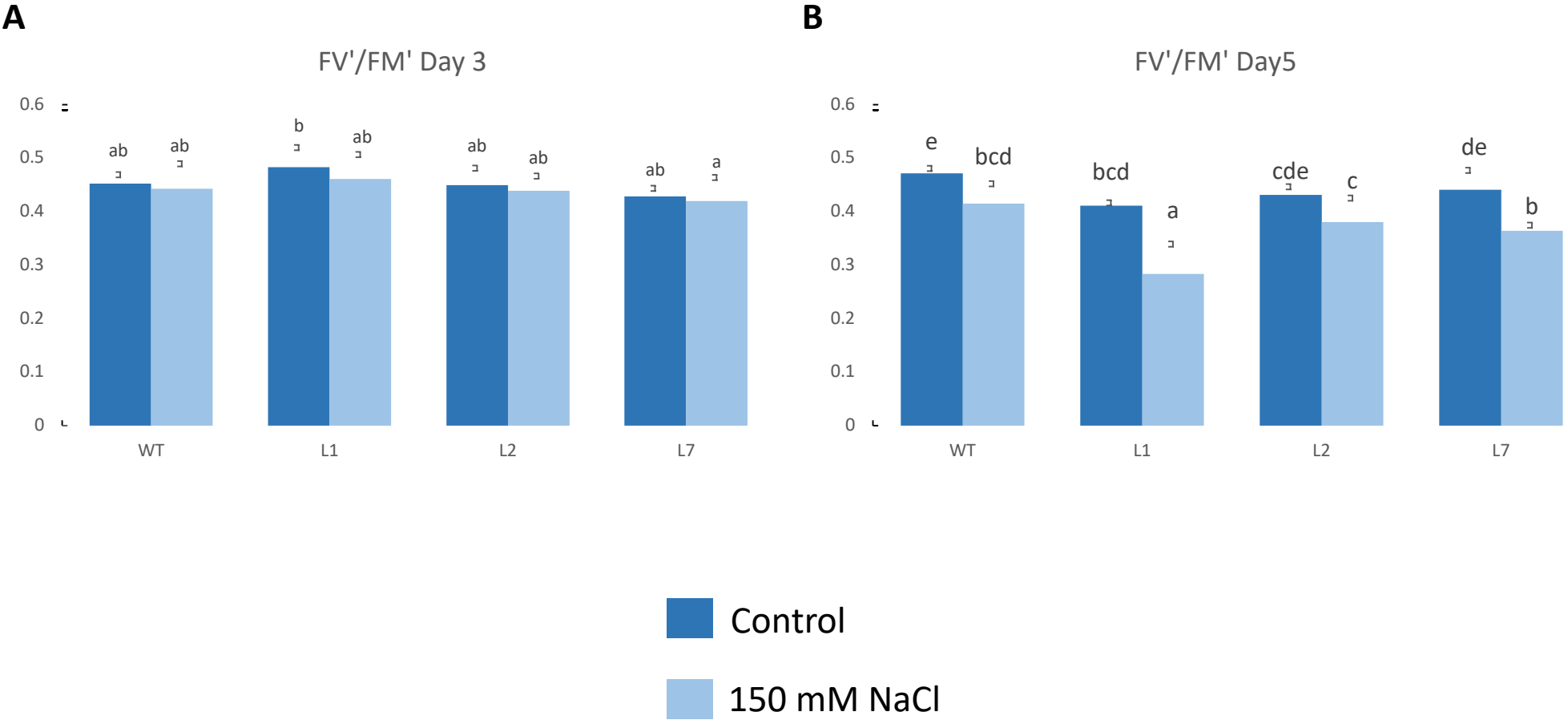

Supplement: Supplementary file 3 — The bar-chart showing FV’/FM’ of the rice(s) under either normal or salt-stress condition. Fluorescence measurement in leaf of the transgenic rice overexpressing OsCam1–1 (L1, L2, L7) and wild type (WT) under normal and salt stress (150 mM NaCl) condition (A) at day 3 and (B) day 5 of treatment. (PDF 31 kb) [file 12870_2018_1538_MOESM3_ESM.pdf]

Figure S3

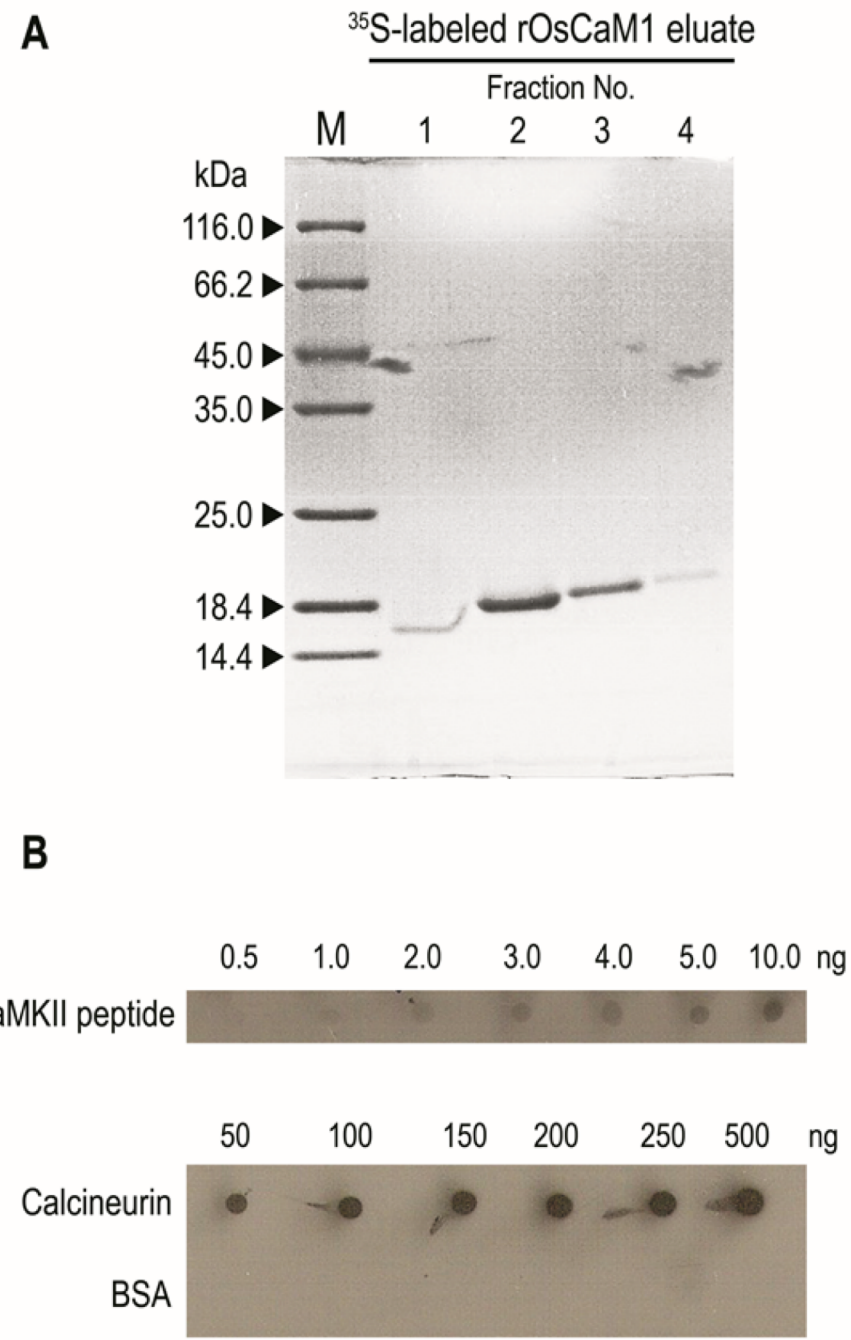

Supplement: Supplementary file 4 — The reliable testing of the 35S-labeled rOsCaM1 binding for testing accuracy and specificity. Examination of the 35S-labeled rOsCaM1 protein, A) 12% SDS-PAGE of the 35S-labeled rOsCaM1 elutes. Lane M: Protein molecular weight marker. Lanes 1–4: Fractions 1–4 of the 35S-labeled rOsCaM1 elute, respectively, and B) Autoradiograph of the blot spotted with various amounts (as written above each spot) of positive (CaMKII peptide and calcineurin) or negative (BSA) control. (PDF 1805 kb) [file 12870_2018_1538_MOESM4_ESM.pdf]

Figure S4

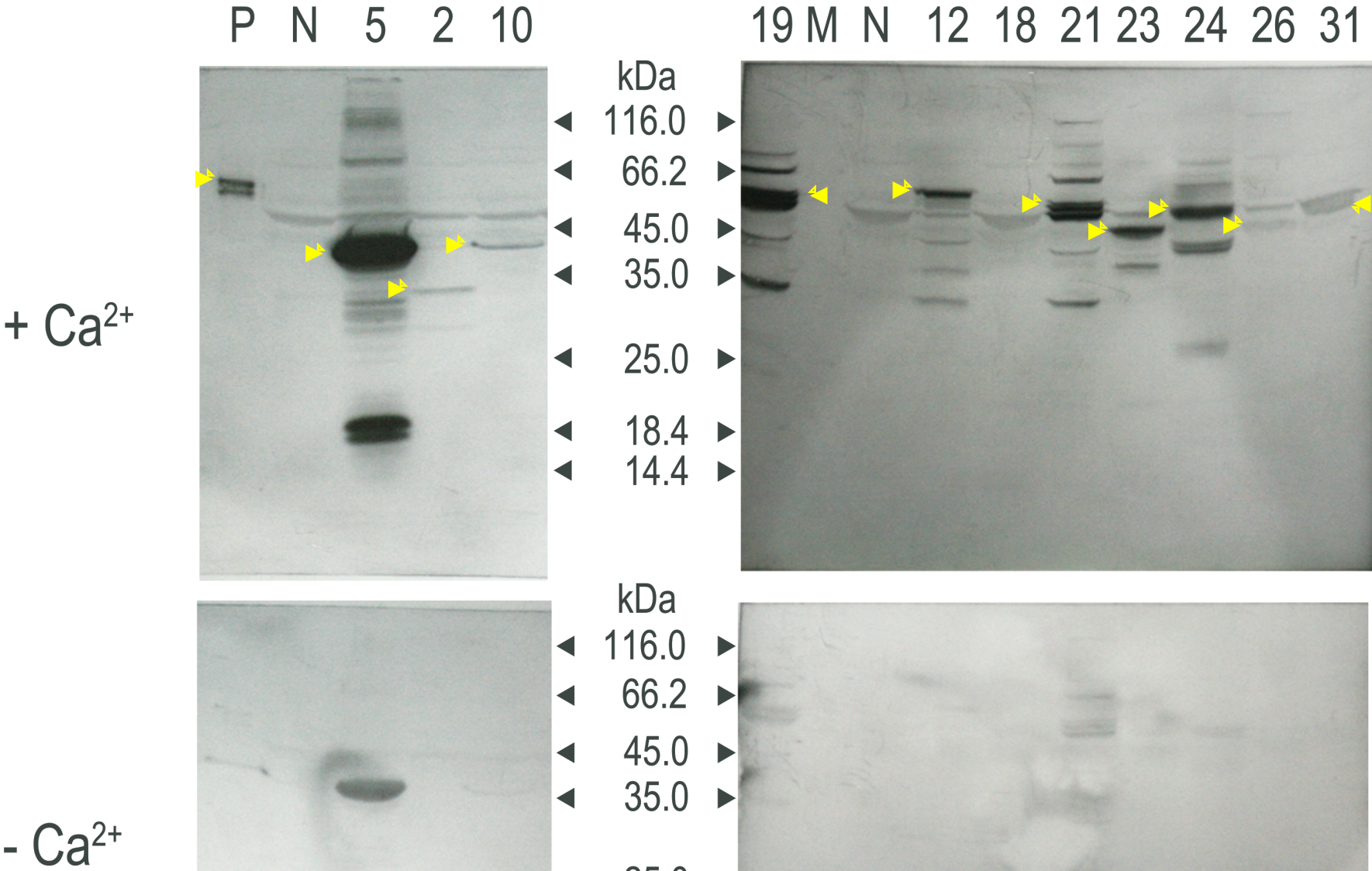

Supplement: Supplementary file 5 — Autoradiographs of far western analysis of 11 putative OsCaM1-binding proteins. Lane M: protein molecular weight markers (negative control); Lane P: calcineurin (positive control); Lane N: crude protein extract from the SOLR cells harboring the pBluescript SK(−) plasmid (negative control); Numbers indicated above the lanes are clone No. assigned when these clones were isolated from the primary screening as following: 2, Cyclic nucleotide-gated ion channel (CNGC); 5, glutamate decarboxylase (GAD); 10, Hydroxyanthranilate hydroxyl cinnamoyltransferase (HHT); 12, CaM-binding transcription activator (CAMTA); 19, Kinesin motor domain- containing protein (KCBP); 21, Kinesin motor domain- containing protein (KCBP); 23, Myosin heavy chain; 24, unknown expressed protein, while 18, 26 and 31 which represent response regulator receiver domain-containing protein (PRR), lipin, N-terminal conserved region family protein, and another clone of Kinesin motor domain- containing protein (KCBP) respectively, were unclear. (PDF 8517 kb) [file 12870_2018_1538_MOESM5_ESM.pdf]
